# Neurotoxic Astrocytes Directly Converted from Sporadic and Familial ALS Patient Fibroblasts Reveal Signature Diversities and miR-146a Theragnostic Potential in Specific Subtypes

**DOI:** 10.3390/cells11071186

**Published:** 2022-04-01

**Authors:** Cátia Gomes, Catarina Sequeira, Shibi Likhite, Cassandra N. Dennys, Stephen J. Kolb, Pamela J. Shaw, Ana R. Vaz, Brian K. Kaspar, Kathrin Meyer, Dora Brites

**Affiliations:** 1 Research Institute for Medicines (iMed.ULisboa), Faculty of Pharmacy, Universidade de Lisboa, 1649-003 Lisbon, Portugal; catia.svgomes@gmail.com (C.G.); catseq95@gmail.com (C.S.); armvaz@ff.ulisboa.pt (A.R.V.); 2 The Abigail Wexner Research Institute, Nationwide Children’s Hospital, Columbus, OH 43205, USA; shibi.likhite@nationwidechildrens.org (S.L.); cassandra.dennys-rivers@nationwidechildrens.org (C.N.D.); brian.kaspar@nationwidechildrens.org (B.K.K.); kathrin.meyer@nationwidechildrens.org (K.M.); 3 Department of Neurology, The Ohio State University Wexner Medical Center, Columbus, OH 43214, USA; stephen.kolb@osumc.edu; 4 Sheffield Institute for Translational Neuroscience (SITraN), University of Sheffield, Sheffield S10 2HQ, UK; pamela.shaw@sheffield.ac.uk; 5 Pharmaceutical Sciences and Medicines, Faculty of Pharmacy, Universidade de Lisboa, 1649-003 Lisbon, Portugal; 6 Department of Pediatrics, College of Medicine, The Ohio State University, Columbus, OH 43210, USA

**Keywords:** amyotrophic lateral sclerosis (ALS), astrocyte-mediated neurotoxicity, fibroblast-transdifferentiated astrocytes, inflammatory-associated miRNAs, miR-146a modulation, patient phenotype subtypes, small extracellular vesicles (sEVs), mutant SOD1 (mSOD1), sporadic ALS (sALS)

## Abstract

A lack of stratification methods in patients with amyotrophic lateral sclerosis (ALS) is likely implicated in therapeutic failures. Regional diversities and pathophysiological abnormalities in astrocytes from mice with SOD1 mutations (mSOD1-ALS) can now be explored in human patients using somatic cell reprogramming. Here, fibroblasts from four sporadic (sALS) and three mSOD1-ALS patients were transdifferentiated into induced astrocytes (iAstrocytes). ALS iAstrocytes were neurotoxic toward HB9-GFP mouse motor neurons (MNs) and exhibited subtype stratification through GFAP, CX43, Ki-67, miR-155 and miR-146a expression levels. Up- (two cases) and down-regulated (three cases) miR-146a values in iAstrocytes were recapitulated in their secretome, either free or as cargo in small extracellular vesicles (sEVs). We previously showed that the neuroprotective phenotype of depleted miR-146 mSOD1 cortical astrocytes was reverted by its mimic. Thus, we tested such modulation in the most miR-146a-depleted patient-iAstrocytes (one sALS and one mSOD1-ALS). The miR-146a mimic in ALS iAstrocytes counteracted their reactive/inflammatory profile and restored miR-146a levels in sEVs. A reduction in lysosomal activity and enhanced synaptic/axonal transport-related genes in NSC-34 MNs occurred after co-culture with miR-146a-modulated iAstrocytes. In summary, the regulation of miR-146a in depleted ALS astrocytes may be key in reestablishing their normal function and in restoring MN lysosomal/synaptic dynamic plasticity in disease sub-groups.

## 1. Introduction

Amyotrophic lateral sclerosis (ALS) is the most common motor neuron (MN) disease in adults, for which there are only limited disease-modifying therapies of modest impact [1,2]. The phenotypic variability observed in ALS and in other neurodegenerative disorders has been a major topic under debate, since it is a key factor contributing to the recurrent failure of clinical trials [3,4,5]. Astrocytes have been extensively described as important players in ALS disease onset and progression [6,7]. Moreover, the astrocyte heterogeneity observed in ALS and other MN diseases [8] may be implicated in the difficulty in finding effective therapies for these disorders. Indeed, induced pluripotent stem cell (iPSC)-based approaches have provided increasing recognition that there are heterogeneous populations of astrocytes, highlighting the existence of subtypes and the need for patient stratification to better understand their specific roles [9,10]. In the present study, we aimed to explore this heterogeneity by using the trans-differentiation process of converting patient fibroblasts into induced astrocytes (iAstrocytes).

The ALS cases are grouped into two different categories: sporadic (sALS) and familial (fALS). Around 5–10% of ALS cases are classified as fALS with a predominantly autosomal dominant pattern of inheritance, while the remaining 90–95% occur sporadically [11,12]. Worldwide, mutations in the C9orf72 gene account for 30–40% of fALS cases [13,14], while mutations in the superoxide dismutase 1 (mSOD1) gene contribute to approximately 20% [15]. Notably, SOD1 mutations account for about 60% of the fALS cases in the Asian population [16,17]. Increased glial activation was observed in spinal cord tissue from both sALS and fALS patients, and neurotoxicity induced by sALS-patient-derived astrocytes was found to be similar to the one of astrocytes from fALS cases [18,19]. These findings support the concept of a glia-based ALS pathophysiology across different forms of the disease. To date, it is still unclear which are the gene/protein signatures of human ALS astrocytes, and the mechanisms implicated in their neurotoxic properties are not fully elucidated. 

A specific aberrant and neurotoxic phenotype was recently described in the cortical and spinal astrocytes from mSOD1 rodents expressing SOD1^G93A^ [20,21], which is the most widely used animal model to study ALS [22]. The reactive profile of such astrocytes is characterized by decreased levels of glial fibrillary acidic protein (GFAP) and glutamate transporter 1 (GLT-1), together with increased expression of S100 calcium binding protein B (S100B) and connexin 43 (Cx43), as well as elevated cell proliferative capacity [20,21]. However, it is not known whether this phenotype is a common feature of all the ALS-patient-derived astrocyte lines, or if such an abnormal phenotype could be responsible for astrocyte-induced MN death in multiple subtypes of ALS. In fact, the translation of these findings to humans has been hampered by limited access to the central nervous system (CNS) tissue samples. This aspect may contribute to the failure of clinical trials, due, in part, to functional differences between rodent and human cells [23]. Most studies indicating the pathogenicity of astrocytes in ALS have been performed in rodent models [24]. Therefore, the use of neural cells differentiated from patient-derived iPSCs, or from induced neural progenitor cells (iNPCs), have become valuable tools for disease modeling and therapeutic testing. The advantages of the direct reprogramming of adult cells over iPSCs are that they can avoid the pluripotent state [25], retain the ageing features of the donor fibroblasts [26], are less contaminated by neuronal cells [27,28], and do not require further purification or selection steps [19]. These characteristics were the rationale for the use of direct conversion into iAstrocytes in the present study. Because this procedure is less time consuming (usually 3–5 weeks) [19] than methods using iPSCs (approximately 24 weeks) [29], it may be particularly relevant for patient stratification and regenerative medicine when considering the short survival of patients with ALS, where the fast generation of astrocytes potentially allows the timely testing of specific therapeutic strategies in the cells of each individual patient. However, such procedure is characterized by the low conversion efficiency from one cell type to another and by the limited cell generation scalability, given the restricted number of post-mitotic cells [25].

Increasing evidence supports the role of microRNAs (miRNAs) in the neuroinflammatory, and neurodegenerative mechanisms linked to ALS [30,31]. However, the use of microarrays does not always allow the detection of miRNA dysregulation in disease pathophysiology, given the limited detection of mature miRNAs due to their small size [32]. In particular, some miRNAs have been defined as inflamma-miRNAs since they are associated with multiple inflammatory pathways [33], such as the trio miR-155, miR-146a and miR-21 [34,35]. We identified an increase in such miRNAs in the lumbar spinal cord of symptomatic mSOD1 mice, while decreased levels of miR-146a and miR-21 were found in the cerebral cortex [20,36]. However, miRNAs are cell-type-specific, and their secretion into the secretome depends on cellular metabolic requirements [37,38,39]. 

We have observed that astrocytes isolated from different CNS regions of the mSOD1 mice are phenotypically different [40], as also highlighted by others [41]. Cortical astrocytes show an increase in the alarmin high mobility group box 1 (HMGB1), together with a decrease in miRNA(miR)-146a, thus supporting the upregulation of the interleukin 1-receptor-associated kinase-1 (IRAK1) and the TNF receptor-associated factor 6 (TRAF6), which represent two of miR-146a targets [20]. In the same study, the decrease in miR-146a was observed in cortical homogenates before and after disease onset, suggesting that astrocytes are the drivers of such depletion in the cerebral cortex. In contrast, miR-146a was not decreased in the spinal cord of the ALS mice, before or after disease onset [36]. Actually, the miR-146a upregulation in the spinal homogenates from the mSOD1 mice at the symptomatic stage, indicates its differential distribution and dependence on the cell environment. Moreover, isolated astrocytes from the spinal cord showed an elevation in miR-146a after 5 days in vitro (DIV) that disappeared at 13 DIV, while miR-155 upregulation was found only in mature spinal astrocytes at 13 DIV [40]. Therefore, the increase in miR-146a in the spinal cord at the symptomatic stage seems to be due to microglia and not to astrocytes, while the depletion in miR-146a is a characteristic of cortical astrocytes in the mSOD1 mice. The increase in miR-155 was found in the spinal microglia and astrocytes, but not in the brain cortex or cortical astrocytes [20]. This is interesting, given that anti-miR-155 was shown to have benefits in attenuating microglia activation and improving the survival of mSOD1 mice [42]; however, no data were provided relatively to the expression of miR-155 in astrocytes. 

The upregulation of miR-146a towards normal levels in mSOD1 cortical astrocytes, counteracted their phenotypic abnormalities, was mirrored in their small extracellular vesicles (sEVs), and prevented secretome-mediated microglia and MN pathological events [43]. In previous studies, we also suggested that such effects may be linked to the paracrine signaling exerted by the transference of miRNAs from the donor cells into their sEVs and shuttling into recipient cells [44,45,46]. However, to what extent these miRNAs are differentially expressed in ALS-patient-derived astrocytes and transmitted in their sEVs has, to date, scarcely been investigated. 

In ALS, there is an urgent need of reliable biomarkers for early diagnosis and patient sub-classification, and as measures of target engagement and therapeutic efficacy [47]. In the present pilot study, we used transdifferentiated astrocytes from patients with sALS and SOD1 mutations (mSOD1-ALS) to verify their neurotoxic nature, and to assess differences in the cell line signatures for astrocyte reactive markers and miRNAs associated with inflammation. Our goal was to validate this in vitro model as suitable for use in patient stratification and in personalized medicine testing. By assessing selective biomarkers identified in cortical astrocytes isolated from the mouse model [20,36,40], we here aimed to validate at least some of them in the human reprogrammed iAstrocytes from the selected ALS patients. iAstrocytes differentiated from iNPCs that were directly converted from mSOD1-ALS and sALS patient fibroblasts were then assessed for astrocyte-associated GFAP, Cx43 and Ki-67, as well as for inflammatory-associated miR-181b, miR-21, miR-155 and miR-146a. Despite the low number of patient cell lines used in this study, we demonstrate that these markers are not similarly expressed by all ALS patient iAstrocytes, providing new insights into patient stratification. We then focused on patients’ lines showing depleted levels of miR-146a. We selected two of the cell lines revealing the most depressed levels of miR-146a, either intracellularly or in their sEVs to be modulated with the miR-146a mimic. Our data revealed that miR-146a downregulation may be a predictive biomarker of specific ALS subtypes and a potential therapeutic target to recover the astrocyte neuroprotective phenotype.

## 2. Materials and Methods

### 2.1. Skin Fibroblast Isolation 

Human skin fibroblast samples were obtained from Stephen J. Kolb (ALS/MND Multidisciplinary Clinic and Translational Research Program, Department of Neurology, The Ohio State University, Wexner Medical Center, Columbus, OH, USA) and from Pamela J Shaw (Sheffield Institute for Translational Neuroscience (SITraN), University of Sheffield, Sheffield, UK). Informed consent was obtained from all subjects before sample collection. Receipt of human tissues was granted through Nationwide Children’s Hospital and Ohio State Institutional Review Boards. Samples included two non-ALS controls and seven ALS patients, three of them carrying SOD1-associated mutations and four with sALS (Table 1). Dissociation of skin biopsies and culture expansion of the fibroblasts was initiated within 30 min post sampling under sterile conditions, as previously described [19,48], in the Brian Kaspar and Kathrin Meyer laboratory at the Center for Gene Therapy at the Nationwide Children’s Hospital, The Ohio State University. In brief, 2 cm^2^ skin samples were washed with phosphate buffered saline (PBS) and placed on a 10 cm^2^ dish with the epidermal side down; 0.5 cm^2^ strips were cut and incubated with 25 mL of 0.05% trypsin/EDTA (Invitrogen Corporation, Carlsbad CA, 92008, USA) in a 50 mL conical tube at 37 °C for 45 min. After that, 20 mL of Dulbecco’s Modified Eagle Medium (DMEM) containing 10% fetal bovine serum (FBS, Gibco/ThermoFisher, Waltham, MA, USA) was added, and cells were centrifuged at 350 g for 4 min. The cell supernatant was aspirated and fresh DMEM containing 10% FBS was added before plating cells in a six-well plate.

### 2.2. Preparation of Retrovirus for Reprogramming

The preparation of the retroviral vectors carrying POU transcription factor Oct-3/4 (Oct3/4), SRY-related HMG-Box Gene 2 (Sox2), Kruppel-like factor 4 (Klf4), and c-Myc was performed as described [49,50], with minor modifications. Briefly, DNA for klf-4, Oct3/4, Sox2 or c-Myc was obtained from Addgene (Cambridge, MA, USA) and transfected, along with packaging plasmids CMV GagPol and CMV VSV-G; these were obtained for retrovirus production in human embryonic kidney carcinoma 293 cells (Hek293T) using the CaCl_2_ transient transfection method. Supernatants were collected for 3 consecutive days beginning 48 h after transfection. Virus concentration was determined using centrifugation of the supernatants at 22,000× *g* for 2 h at 4 °C, followed by resuspension in 0.05M Tris-HCl, 0.1M NaCl and 0.001M EDTA. Viral titers were determined to be approximately 10^8^ viral particles/mL based on immunocytochemical analysis. Human fibroblasts were transduced with retroviruses encoding Oct3/4, Sox2, klf-4 or c-Myc, individually, in human fibroblast media. The concentration of the virus capable of promoting around 70–80% positive cells was selected for each factor, to be used thereafter in the transdifferentiation procedure (Supplementary Figure S1). 

### 2.3. Conversion of ALS Patient Fibroblasts to iNPCs

The direct conversion of ALS patient fibroblasts to iNPCs was carried out according to the methods published by Meyer and colleagues [19,48]. Briefly, one day after being seeded in each well in the six-well plate (an approximate total of 10^5^ cells), skin fibroblasts from patients and non-ALS controls were transduced with a mixture of retroviral vectors expressing Klf4, Oct-3/4, Sox2 and c-Myc (with a multiplicity of infection of 10 for each viral vector). The day after, regular fibroblast medium (DMEM plus 10% FBS) was added. After this recovery time, to promote iNPC conversion, cells were washed with 1X PBS and incubated with fresh medium consisting of DMEM/F12, 1% N2, 1% B27 and containing fibroblast growth factor (FGF, 10 ng/mL, PeproTech, Rocky Hill, NJ, USA), epidermal growth factor (EGF, 10 ng/mL, PeproTech, Rocky Hill, NJ, USA), and heparin (5 μg/mL). This medium was changed every day thereafter. When the cells changed shape and formed sphere-like structures at 7 days post-transduction, indicating that the iNPC culture was established, the medium was switched to iNPC medium consisting of DMEM/F12 (ThermoFisher, Waltham, MA, USA), 1% N2 (Invitrogen, Waltham, MA, USA), 1% B27 (Invitrogen, Waltham, MA, USA), and FGF2 (20 ng/mL). Representative bright-field images of fibroblasts and their direct conversion into iNPCs can be observed in Supplementary Figure S2A.

### 2.4. Differentiation of iNPCs into iAstrocytes

To differentiate iNPCs into iAstrocytes, iNPCs were seeded at low density in a fibronectin-coated 10 cm^2^ plate (human fibronectin, Millipore, Burlington, MA, USA) and cultured with medium containing DMEM, 10% FBS and 0.2% N2. iAstrocytes were then maintained in differentiation/maturation conditions for 6 to 7 days. Representative bright-field images, as well as S100B and GFAP immunocytochemistry of the differentiated iAstrocytes from iNPCs, can be observed in Supplementary Figure S2B,C, which are in accordance with similar images previously published by Meyer and colleagues [19,48]. 

### 2.5. Motor Neuron Differentiation from meSCs

Mouse embryonic stem cells (meSCs) expressing the green fluorescent protein (GFP) under the MN-specific promoter HB9 (HBG3 cells) were a kind gift from Tom Jessell (Columbia University, New York, NY, USA). Their differentiation into MNs was performed as described [19,51]. Briefly, meSCs cells were cultured on a monolayer of EmbryoMax^®^ Primary Mouse Embryonic Fibroblasts (Millipore, Billerica, MA, USA). For differentiation into MNs, cells were lifted with trypsin and resuspended (around 1–2 × 10^6^ meSCs per 10 cm^2^ dish) in culture medium consisting of knockout DMEM/F12, 10% knockout serum replacement, 1% N2, 0.5% L-glutamine, 0.5% glucose (30% in water), and 0.0016% 2-mercaptoethanol) on nonadherent Petri dishes to enable the formation of embryoid bodies (EBs). After one day of recovery, 2 μM retinoic acid (Sigma-Aldrich, St. Louis, MO, USA) and 2 μM smoothened agonist (SAG, a Shh agonist) were added freshly every day with the new medium. After 5 days of differentiation, EBs were dissociated and sorted based on levels of GFP using a FACSVantage/DiVa sorter (BD Biosciences, Rockville, MD, USA). Representative bright field images of meSCs and EBs, as well as immunocytochemical staining for ChAT and Smi-32 MN markers, are depicted in Supplementary Figure S2C.

### 2.6. Assessment of iAstrocyte Neurotoxicity on HB9/GFP-positive MNs

Differentiated iAstrocytes were seeded into a 96-well plate coated with human fibronectin (2.5 μg/mL, Millipore, Burlington, MA, USA) at a density of 10,000 per well. The following day, FACS-sorted GFP-positive MNs were resuspended in medium containing DMEM/F12; 5% horse serum; 2% N2; and 2% B27 plus glial cell-derived neurotrophic factor (GDNF) (Invitrogen, Waltham, MA, USA; 10 ng/mL), brain-derived neurotrophic factor (BDNF) (Invitrogen, Waltham, MA, USA; 10 ng/mL), and ciliary neurotrophic factor (CNTF) (Invitrogen, Waltham, MA, USA; 10 ng/mL); they were then added to the iAstrocytes at a density of 10,000 per well to obtain mixed cell cultures. Each plate was scanned every day with the fully automated IN CELL 6000 confocal plate reader (GE Healthcare, Chicago, IL, USA) to capture GFP-positive cells. After that, we used the IN CELL Analyzer 6000 and the Image Analysis software (GE Healthcare, Chicago, IL, USA) to create whole-well pictures and count MNs, in order to evaluate MN survival. Representative images of the co-cultures are depicted in Supplementary Figure S3.

### 2.7. Immunofluorescence

Cells were fixed with 4% paraformaldehyde (PFA) for 15 min and washed 3 times with Tris-buffered saline (TBS). After that, coverslips were incubated at room temperature (RT) for 1 h with blocking solution consisting of TBS with 10% goat serum, 0.1% Triton X-100, 0.1% Tween-20, and 0.02% sodium azide. Subsequently, cells were incubated overnight at 4 °C with the primary antibodies, all diluted in blocking solution (dilution of antibodies and respective suppliers are listed in Supplementary Table S1). The following day, cells were washed 3 times with TBS before incubation with species-specific fluorescent secondary antibodies and DAPI (for nuclei staining), diluted in blocking solution, for 1.5 h at RT. Finally, coverslips were mounted onto uncoated slides using VECTASHIELD^®^ (Vector Laboratories, Burlingame, CA, USA). Fluorescence images were acquired in an Olympus Bx61 (OLYMPUS CORPORATION, Shinjuku, Tokyo, Japan) microscope or in the fully automated IN CELL 6000 confocal plate reader.

### 2.8. Gene and miRNA Expression Profiling by RT-qPCR

Total RNA was extracted from iAstrocytes using TRIzol^®^ Reagent (Life Technologies, Carlsbad, CA, USA), as described [20]. Determination of mRNA expression was performed by RT-qPCR in our laboratory, as usual [46]. Total RNA was quantified using the Nanodrop ND-100 Spectrophotometer (NanoDrop Technologies, Wilmington, DE, USA) and the conversion to cDNA was performed with Xpert cDNA Synthesis Mastermix (GRiSP, GK81.0100, Porto, Portugal). RT-qPCR was performed in the QuantStudio 7 Flex Real-Time PCR System (Applied Biosystems, Waltham, MA, USA) using Xpert Fast SYBR Mastermix (GRiSP, Porto, Portugal), under optimized conditions: 50 °C for 2 min, followed by 95 °C for 2 min, and finally, 40 cycles at 95 °C for 5 sec, and 62 °C for 30 sec, to determine the mRNA expression of the genes indicated in Supplementary Table S2. To verify the specificity of the amplification, a melt-curve analysis was conducted immediately after the amplification protocol. Non-specific products of PCR were not found in any case. β-actin and GAPDH (average value) were used as endogenous controls to normalize the expression levels.

For miRNA quantification, total RNA was extracted with QIAzol Lysis Reagent (QIAGEN Strasse 1, Hilden, Germany) and the miRNeasy Mini Kit (QIAGEN Strasse 1, Hilden, Germany), and the cDNA conversion was performed using the universal cDNA Synthesis Kit (QIAGEN Strasse 1, Hilden, Germany), as described [36]. For RT-qPCR, Power SYBR™ Green PCR Master Mix (Applied Biosystems, Waltham, MA, USA) was used in combination with the pre-designed primers (QIAGEN Strasse 1, Hilden, Germany). U6 (reference gene) was applied as an endogenous control to normalize the expression levels. The reaction conditions consisted of polymerase activation/denaturation and well-factor determination at 95 °C for 10 min; this was followed by 50 amplification cycles at 95 °C for 10 s, and at 60 °C for 1 min (ramp-rate of 1.6°/s). Primer sequences are listed in Supplementary Table S2. Relative mRNA/miRNA concentrations were calculated using the ΔΔCT equation.

### 2.9. Protein Analysis Using Western Blot

Extraction of proteins from iAstrocyte samples at Meyer and Kaspar laboratory at Nationwide Children’ Hospital (Ohio, USA) was obtained by lysing cells using a radioimmunoprecipitation assay (RIPA). Briefly, protein extracts were separated on an Invitrogen™ NuPAGE™ Novex™ 4–12% Bris-Tris protein gel (Life Technologies, Carlsbad, CA, USA) and transferred to a PVDF membrane (LC2005, Invitrogen, Waltham, MA, USA) previously hydrated with methanol for at least 1 min. After that, membranes were blocked for 1 h at RT with Odyssey Blocking Buffer (Cat No. 927-40000, LI-COR Biosciences, Lincoln, NE, USA), then incubated overnight at 4 °C with primary antibodies diluted in 1:1 blocking solution and TBS with 0.1% Tween-20. The following day, membranes were washed and incubated with fluorescent species-specific secondary antibodies from LI-COR at RT for 1 h and protected from light. Dilution of antibodies and providers are indicated in Supplementary Table S1. After incubation, membranes were washed, and relative intensities of the protein bands were analyzed using the Image Studio Lite Ver 5.2 analysis software (LI-COR Biotechnology, Lincoln, NE, USA), after scanning in the LI-COR, ODYSSEY CLx Imaging System (LI-COR Biotechnology, Lincoln, NE, USA). Results were normalized relative to the expression of β-actin and indicated as fold change. 

For protein analysis after pre-miR-146 modulation in the Brites laboratory at the Research Institute for Medicines (Lisbon, Portugal), lysis and protein isolation were performed as we published [20], and a protein assay kit (Bio-Rad, Hercules, CA, USA) was used to determine protein concentrations. Then, 50 μg of protein was used in an SDS-PAGE and transferred to a nitrocellulose membrane. After blocking with 5% (*w*/*v*) non-fat milk solution, nitrocellulose membranes were incubated overnight at 4 °C with primary antibodies (indicated in Supplementary Table S1). The following day, secondary antibodies conjugated to horseradish peroxidase were used (Supplementary Table S1). The chemiluminescent detection was performed after membrane incubation with WesternBright Sirius (Advansta, San Jose, CA, USA). The relative intensities of protein bands were analyzed using the iBright Analysis software, Desktop Version, after scanning with iBright FL1500 Imaging System (Thermo Fisher Scientific, Rockford, IL, USA). Results in cells were normalized to β-actin and indicated as fold change. 

### 2.10. Isolation and Evaluation of Soluble miRNAs

Small RNAs were isolated from the sEV-depleted conditioned media of iAstrocytes based on the miRNeasy Serum/Plasma Advanced Kit (Qiagen, Hilden, Germany), according to the manufacturer’s instructions. Briefly, approximately 200 µL of astrocyte-conditioned media was mixed with the RPL buffer, and then with RPP buffer. After centrifugation, the supernatant was transferred to a new reaction tube and mixed with isopropanol, and then placed on a RNeasy UCP MinElute column. After centrifugation, the RWT buffer was added to the column, and after a new spin, RPE was placed on the column. After centrifugation, 80% ethanol was added to the column and centrifuged at full speed to dry the membrane. Finally, RNase-free water was added to the RNeasy UCP MinElute column to elute the RNA. Total RNA was quantified using a Nanodrop ND-100 Spectrophotometer (NanoDrop Technologies, Wilmington, DE, USA) and conversion to cDNA was performed using the universal cDNA Synthesis Kit (QIAGEN, Hilden, Germany). For RT-qPCR, Power SYBR™ Green PCR Master Mix (Applied Biosystems, Waltham, MA, USA) was used in combination with pre-designed primers (QIAGEN, Hilden, Germany), as described before for cell miRNAs.

### 2.11. Isolation and Characterization of sEVs

sEVs were isolated from the extracellular media of iAstrocytes, as previously described [52]. Briefly, after 24 h incubation in medium with FBS previously depleted in sEVs, cell supernatant was centrifuged at 1000 *g* for 10 min to pellet the cell debris. Then, the supernatant was transferred to another tube and centrifuged at 16,000× *g* for 1 h to pellet the large extracellular vesicles. After that, the recovered supernatant was filtered in a 0.22 µm pore filter and further centrifuged in an Ultra L-XP100 centrifuge (Beckman Coulter Inc., Brea, CA, USA) at 100,000× *g* for 2 h to pellet the sEVs (size ~30 to 200 nm). The pellet of sEVs was then washed in PBS and centrifuged again at 100,000× *g* for 2 h. All centrifugations were performed at 4 °C. 

The size distribution and concentration of sEVs were assessed with nanoparticle tracking analysis (NTA) using the NanoSight, model LM10-HSBF (Malvern Instruments, Malvern, UK). Samples were injected into the system under controlled flow using a NanoSight syringe pump and integrated scripting control system. Five different videos up to 60 s long were made and particle movement was analyzed using NTA software (version 3.1), as we mentioned before [45]. For the analysis of sEV cargo in terms of miRNAs, the final pellet was resuspended in QIAzol Lysis Reagent (QIAGEN, Hilden, Germany) and the exosomal RNA extracted using the miRNeasy Mini Kit (QIAGEN, Hilden, Germany), according to manufacturer’s instructions, and as described above.

### 2.12. Upregulation of miR-146a in iAstrocytes

To assess the effect of miR-146a upregulation in iAstrocytes, cells were transfected with the pre-miR^TM^ precursor 146a-5p. Briefly, after 5 days of differentiation, iAstrocytes were plated at a low density for transfection. The following day, iAstrocytes were transfected with 20 nM pre-miR^TM^ miRNA precursor 146a (AM17100, Ambion^®^, Thermo Scientific, Waltham, MA, USA) or with 20 nM pre-miR™ miRNA Precursor Negative Control (AM17110, Ambion^®^, Thermo Scientific, Waltham, MA, USA) mixed in X-tremeGENE™ HP DNA Transfection Reagent (Sigma-Aldrich, St. Louis, MO, USA) and diluted in Opti-MEM™ (Thermo Scientific, Waltham, MA, USA). After approximately 10 h incubation, cells were washed with PBS, and fresh astrocyte medium was added (DMEM supplemented with 1% FBS (exosome-depleted), 0.2% N2, and 1% Antibiotic-Antimycotic solution). Cells were collected after 24 h and used in the next experiments. Results were analyzed in the iAstrocytes treated with the pre-miR-146a, relative to the respective cell line treated with pre-miR negative control.

To assess whether the transfection method or miR-146a upregulation affected iAstrocyte viability, phycoerythrin-conjugated Annexin V (Annexin V-PE) and 7-amino-actinomycin D (7-AAD; Guava Nexin^®^ Reagent, no.4500-0450, Millipore, Burlington, MA, USA) were used to determine the percentage of viable cells (Annexin V-PE and 7-AAD negative) using flow cytometry [53]. 

### 2.13. iAstrocytes and NSC-34 MN Co-cultures

iAstrocytes transfected with the pre-miR-146a, or treated with the pre-miR negative control, were co-cultured with the NSC-34 MN-like cells to assess the effects produced by the modulated iAstrocytes, as described before [20]. Briefly, NSC-34 cells expressing the WT human SOD1 (WT MNs) were maintained for 48 h in the proliferation media (DMEM high glucose and without pyruvate, supplemented with 10% FBS and 1% penicillin/streptomycin, and geneticin sulfate (G418, 0.5 mg/mL) for selection), followed by an additional 48 h in the differentiation media (DMEM-F12 plus FBS (1%), nonessential amino acids (1%) and penicillin/streptomycin (1%)), as we described before [54]. iAstrocytes were seeded onto paraffin-dot-containing coverslips [42], which were placed on top of a layer of NSC-34 MNs, in the proportion of 1:1. Cells were co-cultured for 72 h, then harvested separately. We used the NSC-34 cells in the present work—in our laboratory, as usual—based on our extensive experience with this human cell line, on their effectiveness in revealing neurotoxic events by reactive astrocytes [20,40,43,54], and because MNs differentiated from embryonic stem cells or iPSCs have an immature and not-reproducible phenotype [55,56].

The expression of BECN1 (gene that encodes for beclin-1), SYP (gene that encodes for synaptophysin), DLG4 (gene that encodes for post-synaptic protein 95, PSD95), KIF5B (gene that encodes for the kinesin-1) and DYNC1H1 (gene that encodes for dynein), as well as of miR-146a, was determined using RT-qPCR in NSC-34 MNs, as described above. The list of primers used in this study is indicated in Supplementary Table S2. 

For lysosome and autophagosome labelling, MNs were incubated with 50 nM LysoTrackerTM Red (L7528, Invitrogen, Waltham, MA, USA) for 30 min at 37 °C, then fixed with 4% PFA. Cell nuclei were stained with Hoechst 33258 dye. Images were acquired using an AxioCam HR camera adapted to an AxioScope A1 microscope (Zeiss, Germany). Images were then analyzed using the ImageJ software.

### 2.14. Statistical Analysis

Results of at least three independent experiments were expressed as mean values ± SEM. Statistical analyses were performed using one-way ANOVA followed by Dunnett’s Multiple Comparison post hoc test for mean differences, relative to the average of the directly converted control lines (CTR1 and CTR2). Differences between iAstrocytes transfected with pre-miR-146a and respective iAstrocytes treated with the pre-miR negative control were accessed using a two-tailed paired Student’s *t*-test. Statistical analysis was performed using GraphPad PRISM 5.0 (GraphPad Software, San Diego, CA, USA). *p* < 0.05 was considered statistically significant.

## 3. Results

### 3.1. iAstrocytes Derived from Patients with ALS Show Neurotoxic Properties 

In this study, our first interest was to validate that iAstrocytes differentiated from iNPCs generated by direct conversion from four sALS patients and three mSOD1-ALS cases were toxic for HB9-positive MNs after 96 h in co-culture, as previously described by Meyer et al. [19]. We used iAstrocytes directly converted from fibroblasts, as previously published by Meyer et al. [19] and Dennys et al. [48], collected from such ALS patients (three males and four females) and two non-ALS controls (one male and one female) ranging in age from 29 to 69 years-old, after informed consent (Table 1). 

**Figure 1:**
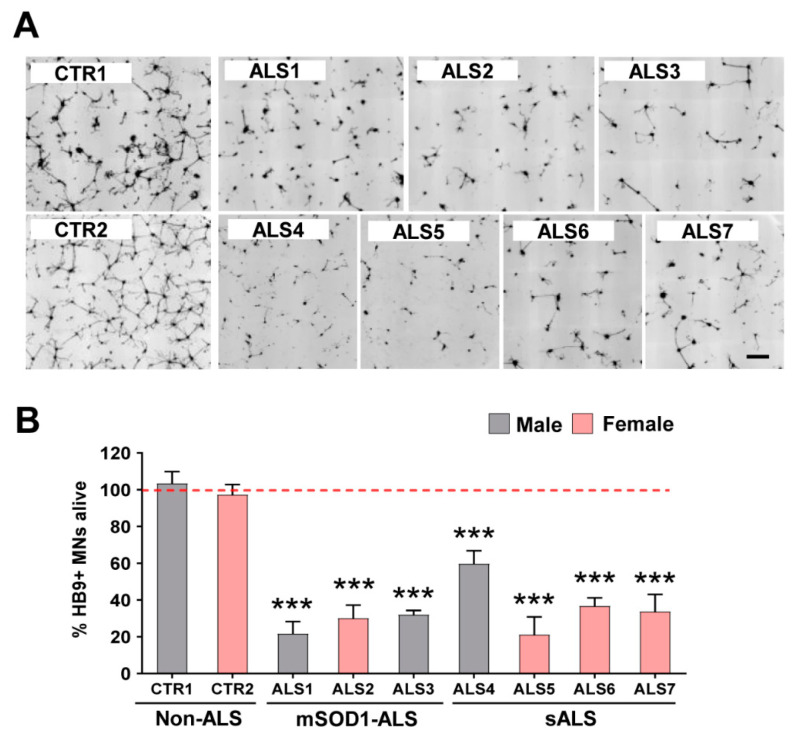
Viability of motor neurons (MNs) is drastically reduced in the presence of iAstrocytes from patients with amyotrophic lateral sclerosis (ALS), either sporadic (sALS) or with SOD1 mutations (mSOD1-ALS). Induced astrocytes (iAstrocytes) were differentiated from induced neural progenitor cells obtained by direct conversion from patient fibroblasts, as described in Methods. Such iAstrocytes were co-cultured with HB9/GFP-positive MNs for 96 h. (**A**) Representative images of MNs (in black) from the seven ALS cases showing a lower number of viable cells as compared to the two controls (CTR1 and CTR2); (**B**) MN survival percentage expressed as mean values ± SEM from at least five independent experiments. *** *p* < 0.001 vs. average controls, one-way ANOVA followed by Dunnett’s post hoc test. Scale bar represents 200 µm.

Mouse HB9/GFP-positive MNs were cultured with iAstrocytes for 96 h (Supplementary Figure S3), and their survival monitored by daily confocal image acquisition, as described in Methods. After starting to extend neurites at 24 h, we observed that cells started to die, and the survivors exhibited shorter neurites. As expected, at 96 h, iAstrocytes isolated from either mSOD1-ALS or sALS patients, independently of the sex, caused a marked reduction in neurites, compared to the controls (Figure 1A,B). Moreover, only 20–60% of the cells survived in those co-culture conditions (*p* < 0.001), highlighting the neurotoxic properties of ALS-patient-derived iAstrocytes. The two most toxic cases, ALS1 and ALS5, were found in sALS and fALS groups, respectively. The less noxious iAstrocytes were derived from the fibroblasts of the younger sALS4 patient with the bulbar onset form. 

### 3.2. iAstrocytes from mSOD1-ALS and sALS Patients Show Distinct Cell Subtype Signatures 

Astrocyte reactivity and astrogliosis were identified in ALS patient tissue samples and are suggested to contribute to MN degeneration [57]. Importantly, iPSC-based approaches have provided growing recognition that there are heterogeneous populations of astrocytes supporting the existence of subtypes, and that patient stratification is needed to better understand their specific roles [9,10]. Therefore, despite the similar neurotoxic properties shown by all the ALS patient cell lines, we next studied the potential signature differences. We focused our evaluation on the known markers of cell reactivity/aberrancy and inflamma-miRNAs, previously identified in ALS models [20,21,30,58]. 

**Figure 2:**
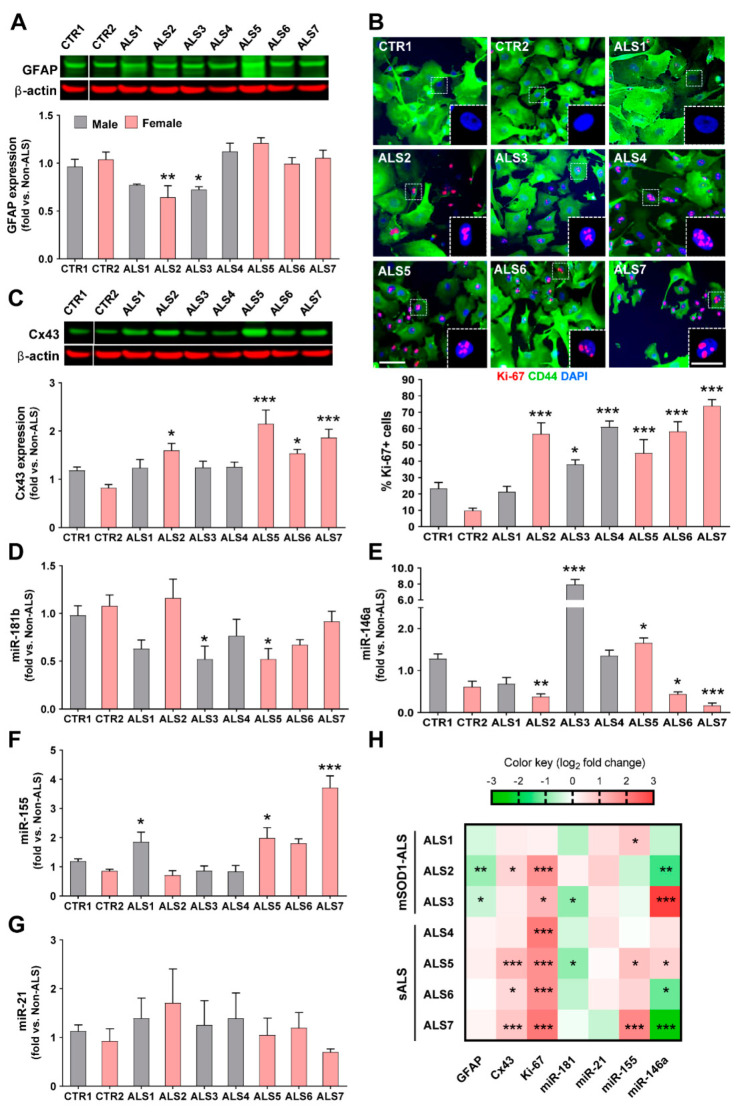
Reactive markers and inflammatory miRNAs are distinctly expressed by iAstrocytes from patients with amyotrophic lateral sclerosis (ALS), either sporadic (ALS4–ALS7) or with SOD1 mutations (ALS1–ALS3). Induced astrocytes (iAstrocytes) were differentiated from induced neural progenitor cells obtained by direct conversion from patient fibroblasts, as described in Methods. (**A**) Decreased GFAP expression assessed by Western blot analysis was only observed in two mSOD1-ALS cases; (**B**) representative images of Ki-67 staining in iAstrocytes with fluorescent puncta in the nuclei (pink, insets). Proliferation of iAstrocytes was observed in six out of seven patients and determined by Ki-67 staining, and the number of cells with positive nuclear Ki-67 (pink) was counted, as described in Methods. Cell cytoplasm was stained with the astrocyte marker CD44 (green). DAPI (blue) was used for nuclear staining; (**C**) connexin 43 (Cx43) was increased in four samples and was assessed using Western blot analysis in iAstrocytes. Representative results from one blot in which samples ran in the same gel with the same conditions are shown for GFAP and Cx43. Results are mean values ± SEM fold change vs. non-ALS controls from at least five independent experiments; expression of (**D**) miRNA (miR)-181b, (**E**) miR-146a, (**F**) miR-155, and (**G**) miR-21 in iAstrocytes, determined by RT-qPCR, reveals variability among patients, with three patients showing elevated miR-155 levels and an identical number revealing decreased miR-146a or miR-181b. Results are mean values ± SEM fold change vs. non-ALS controls from at least three independent experiments; (**H**) heatmap summary of the reactive markers and inflammatory-associated miRNAs in iAstrocytes for comparative analysis of sALS and mSOD1-ALS patient signatures. * *p* < 0.05, ** *p* < 0.01 and *** *p* < 0.001 (compared with the average taken from control lines: CTR1 and CTR2), one-way ANOVA followed by Dunnett’s post hoc test; SOD1—superoxide dismutase 1. Scale bars represent 50 μm and 20 μm (insets).

Although all cells were toxic to MNs, we observed that the iAstrocyte phenotypes, based on a set of reactive markers and miRNAs, were different among the assessed ALS patients. Though GFAP upregulation is usually associated with astrocyte proliferation and reactive astrogliosis [59], no increased expression was noticed in any of the samples; however, a significant downregulation was found in two of the mSOD1-ALS patients, one female and one male (ALS2 and ALS3, *p* < 0.01 and *p* < 0.05, respectively, Figure 2A).

Astrocyte proliferation is also a feature associated with astrogliosis and astrocyte aberrancy [20,21], and interestingly, most of the ALS-iAstrocytes showed increased proliferative capacity when compared to the non-ALS controls. Only one of the patients (ALS1) did not show an elevated proliferative index (Figure 2B). The remaining patients presented a high number of Ki-67-positive cells (all *p* < 0.001, except ALS3 with *p* < 0.05). Cx43, another marker of astrocyte reactivity, was found to be upregulated in the spinal cord of symptomatic mSOD1 mice and their neonatal cortical astrocytes [20], as well as in ALS iPSC-derived neurotoxic astrocytes [60]. Here, we verified that it was increased in all iAstrocytes from female patients (at least *p* < 0.05, Figure 2C). 

Dysregulated miRNAs in both fALS and sALS cases were shown to be involved in neuroinflammatory and neurodegenerative pathways [30,58]. The profile of reduced miR-181b in ALS3 and ALS5 samples (at least *p* < 0.05, Figure 2D), was unique relative to the other miRNA profiles (Figure 2E–G), and both patients presented increased levels of miR-146a (at least *p* < 0.05, Figure 2E). The ALS3 patient harboring the mutant SOD1A4V manifested the highest expression of miR-146a.

From all the samples, iAstrocytes from patient ALS5 were those showing the highest number of simultaneous alterations, including the concomitant increase in miR-146a and miR-155 (see comparative heatmap in Figure 2H). Interestingly, it deserves to be noted that among the different cell lines, ALS2, ALS6 and ALS7 stood out by revealing diminished values of miR-146a (*p* < 0.01, *p* < 0.05 and *p* < 0.001, respectively, Figure 2E). The upregulation of miR-155 described for ALS microglia [42] was found to only be overexpressed in the ALS1, ALS5 and ALS7 cell lines (at least *p* < 0.05, Figure 2F). Finally, we did not observe differences for the expression of miR-21 in any of the iAstrocytes derived from patients (Figure 2G). It is worth highlighting that despite showing some similarities and differences in their signatures (Figure 2H), no specific association could be established with sALS or fALS cases. Because only a low number of samples were evaluated, this type of correlations should be further explored in studies analyzing a higher number of patients. However, most of the studies with patient cells also involved few cell lines and had a scientific impact [19,29,61,62,63]. The phenotypic variability observed in the present study between ALS iAstrocyte lines suggests that it may be useful in the stratification and subcategorization of patients. 

Considering our prior data pointing to depressed levels of miR-146a in cortical astrocytes isolated from the mSOD1 mice [20,40]—as well as the potential benefits of its regulation [43], together with its increase in the spinal cord of ALS mice in the symptomatic stage [36]—we selected ALS2, ALS3, ALS6 and ALS7 for the subsequent studies directed at paracrine signaling and miRNA modulation, due to their miR-146a deregulation.

### 3.3. Dysregulated miR-146a Levels in ALS iAstrocytes Are Mimicked in sEV-Free Secretomes and sEV Cargoes, but the Same Is Not True for miR-155

The release of miRNAs into the cell secretome modifies the function of the target cells. We showed that depleted miR-146a in cortical astrocytes isolated from the mSOD1 mice were recapitulated in their released sEVs in the secretome, leading to microglia activation and MN degeneration [40,43]. miRNAs are considered as paracrine signaling mediators, mainly in cancer, and miRNA-based therapy is promising in neurodegenerative diseases [64]. Therefore, based on our previous data on miR-146a dysregulation in ALS astrocytes from the mSOD1 mouse model, we decided to analyze the distribution of miR-146a in the secretome from iAstrocytes differentiated from ALS patients, which revealed alterations in its expression, i.e., ALS2, ALS3, ALS6 and ALS7. Moreover, because the dysregulation of miR-155, in addition to that of miR-146, was also identified in ALS rodent models and patients [42,65,66], we assessed its representation in the secretome of the selected cell lines. For that, we collected the cell secretome and isolated the sEV-free and the sEV fractions, as previously described in Materials and Methods. 

**Figure 3:**
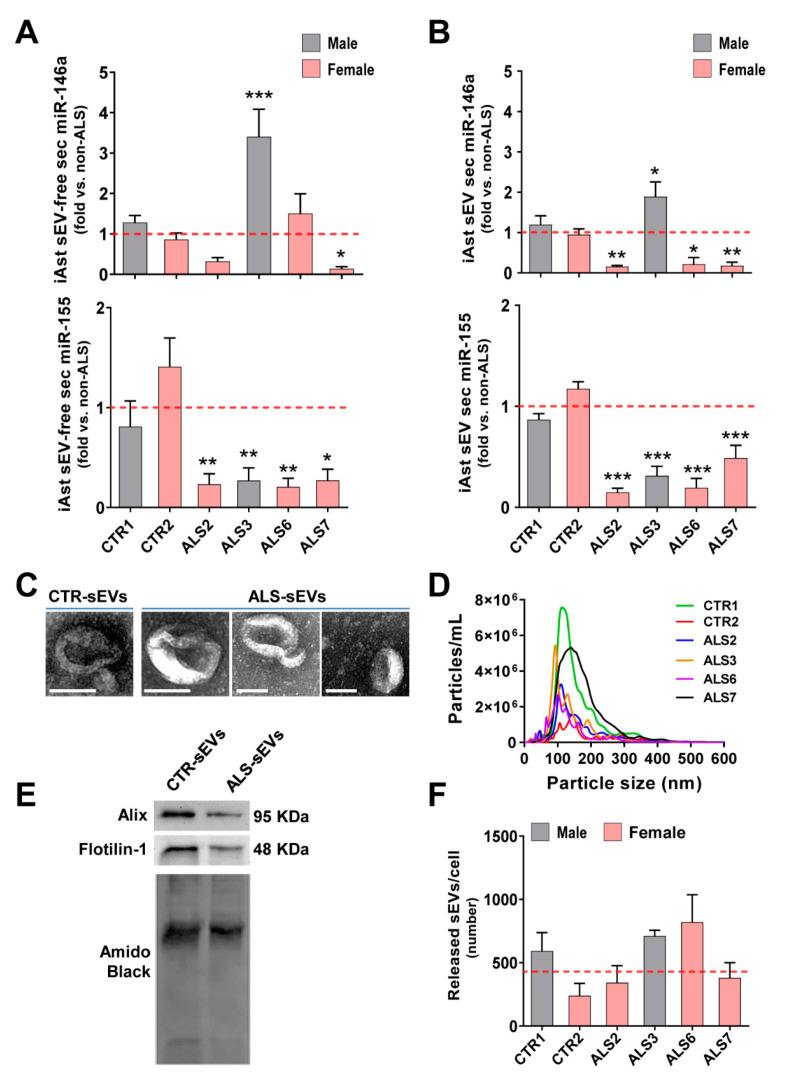
Dysregulated levels of miR-146a in iAstrocytes derived from SOD1-ALS and sALS patient fibroblasts were recapitulated in their secretome, either in sEV-free or in sEV fractions, while intracellular increase in miR-155 was retained and determined low secreted levels. ALS patient fibroblasts were directly transdifferentiated into neural progenitor cells and subsequently differentiated into induced astrocytes (iAstrocytes) for 7 days in vitro. Soluble miRNAs (sEV-free) and small extracellular vesicles (sEVs) were isolated from the extracellular media of mSOD1 (ALS2 and ALS3) and sALS (ALS6 and ALS7) iAstrocytes, as described in Methods. (**A**) Expression of miRNA(miR)-155 and miR-146a in sEV-free secretome of iAstrocytes accessed by RT-qPCR; (**B**) expression of miRNA (miR)-155 and miR-146a in sEVs of iAstrocyte secretomes accessed by RT-qPCR; (**C**) representative images of sEVs using transmission electron microscopy (TEM); (**D**) concentration (particles/mL) and size distribution of sEVs using nanoparticle tracking analysis (NanoSight); (**E**) representative image of common protein markers of sEVs using Western blot analysis; (**F**) comparative average number of released sEVs by each iAstrocyte cell and ALS sample assessment. Results are mean values ± SEM fold change vs. non-ALS controls (CTR1 and CTR2) from at least three independent experiments. * *p* < 0.05, ** *p* < 0.01 and *** *p* < 0.001 vs. non-ALS controls (average from CTR1 and CTR2), one-way ANOVA followed by Dunnett’s post hoc test. ALS—amyotrophic lateral sclerosis; iAst—iAstrocytes; sALS—sporadic ALS; mSOD1—mutant superoxide dismutase 1; sec—secretome. Scale bar represents 100 nm.

We found that the increased levels mainly exhibited by the ALS3 iAstrocytes were recapitulated in their sEV-free secretome (*p* < 0.001, Figure 3A) and isolated sEVs (*p* < 0.05, Figure 3B). Most interestingly, the low intracellular levels of miR-146a in ALS2, ALS6 and ALS7, were recapped in sEVs for ALS2 and ALS6 (at least *p* < 0.05) and in both sEV-free and sEV fractions for ALS7 (at least *p* < 0.05). Then, we investigated the paracrine signaling associated with miR-155 in those samples. To our surprise, despite the elevated levels of miR-155 in ALS7 and ALS5 cell lines, their secretomes revealed depressed levels of miR-155, either in their sEV-free secretomes (at least *p* < 0.05, Figure 3A) or in the isolated sEVs (*p* < 0.001, Figure 3B). 

Our data revealed that the iAstrocyte secretome recapitulated the miR-146a levels found in cells, but that the expression of miR-155 was not the same in cells and in sEV-free secretome or sEVs. Therefore, upon modulation, it was predicted that the miR-146a mimic would normalize its levels in the secretome. As predicted, and in agreement with previous data in cortical astrocytes isolated from the mSOD1 mice, pre-miR-146a treatment efficiently recovered the normal levels in the secretome [43]. In contrast, if we regulated the overexpressed miR-155 with the inhibitor, it would further decrease the already low levels of miR-155 in both secretome fractions. 

We were particularly interested in the content of miRNAs in sEVs, since they are promising biomarkers and potential delivery carriers of miRNA mimics and inhibitors [37,67,68,69]. In fact, miRNA inclusion in sEVs is more stable than its free species [68], and sEVs are known to travel longer distances and to cross the blood–brain barrier [70]. Therefore, we next characterized sEVs from the controls and ALS iAstrocytes. As expected, sEVs showed the characteristic round and cup-shaped morphology at high magnification using transmission electron morphology (TEM); a predominant ~100–150 nm particle diameter using nanoparticle tracking analysis; and the typical proteins Alix and Flotilin-1 using Western blot (Figure 3C–E), as we described [45]. It is worth noting, however, that a large range size was noticed in sEVs from the ALS7 sample, but not for the number of released vesicles when compared to all the other samples, either from controls or from patients (Figure 3F). More than a 2-fold increase in the number of sEVs released by ALS6 iAstrocytes after a 24 h incubation was noticed, though it was not significant. Therefore, no compromised release of sEVs was noticed among the analyzed samples.

### 3.4. Upregulation of miR-146a in Depleted ALS2 and ALS7 iAstrocytes Differently Regulates Its Targets and Reactive Biomarkers

Based on the data achieved in the previous section, we chose to only focus our study on the modulation of miR-146a with the ALS2 and ALS7 cell lines—i.e., those with the lowest expression of miR-146a, and corresponding to a case with a SOD1 mutation and to a sporadic one, respectively. The decision was based on the benefits that we obtained with the miR-146a mimic in mSOD1 mouse cortical astrocytes towards the recovery of their neuroprotective phenotype [43], and considering our interest in reestablishing cell homeostatic balance through the secretome recapitulating cell miRNA levels, only observed for miR-146a. 

**Figure 4:**
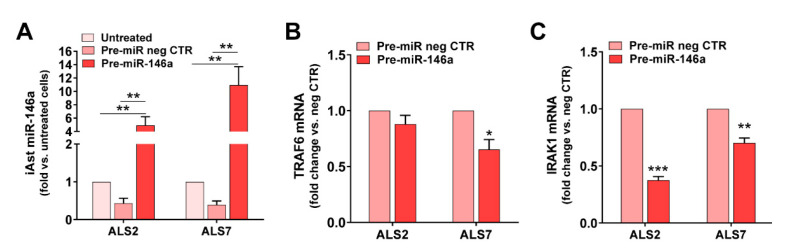
Transfection with pre-miR-146a leads to its upregulation in depleted ALS-iAstrocytes, while reduces the expression of TRAF6 and IRAK1, which are known targets of miR-146a. ALS patient fibroblasts were directly transdifferentiated into induced neural progenitor cells, and subsequently differentiated into induced astrocytes (iAstrocytes, iAst) for 7 days in vitro. For miRNA(miR)-146a upregulation, mSOD1-ALS2 and sALS7 iAst were transfected with pre-miR-146a. (**A**) Expression of miR-146a in the non-treated iAst and after treatment with the pre-miR negative control (pre-miR neg CTR) or pre-miR-146a using RT-qPCR; (**B**) expression of the TNF-receptor-associated factor 6 (TRAF6) in the non-treated iAst and after treatment with the pre-miR neg CTR or pre-miR-146a by RT-qPCR; (**C**) expression of the interleukin 1-receptor-associated kinase 1 (IRAK1) in the non-treated iAst and after treatment with the pre-miR neg CTR or pre-miR-146a using RT-qPCR. Results are mean values ± SEM fold change vs. untreated cells (in (**A**)) and transfected with pre-miR neg CTR (in (**B**,**C**)), from at least four independent experiments. ** *p* < 0.01, one-way ANOVA followed by Tukey’s post hoc test (in (**A**)), or * *p* < 0.05, ** *p* < 0.01 and *** *p* < 0.001 vs. respective cells treated with pre-miR neg CTR, paired Student’s *t*-test (**B**,**C**). ALS—amyotrophic lateral sclerosis; sALS—sporadic ALS; mSOD1—mutant superoxide dismutase 1.

In conformity, we tested pre-miR-146a modulation in the selected patient iAstrocytes and used either the untreated cells or the cells treated with the pre-miR negative CTR as controls (Figure 4). We assessed the success of the transfection and the miR-146a regulation of their targets IRAK1 and TRAF6, both inducers of the TLR/NF-kB inflammatory pathway and associated with miR-146a anti-inflammatory properties [71]. Transfection with pre-miR-146a increased its expression by ~5 times in ALS2 and ~11 times in the ALS7 iAstrocytes vs. untreated or transfected cells with the pre-miRNA negative control (*p* < 0.01, Figure 4A). Such treatment did not cause any alteration in cell viability (Supplementary Figure S4). While the treatment of iAstrocytes with the pre-miR-146a caused a decrease in the TRAF6 mRNA in the ALS7 cells (*p* < 0.05), but not in ALS2, it led to common downregulated levels of IRAK mRNA levels in both ALS2 and ALS7 iAstrocytes (at least *p* < 0.01), when compared with the corresponding cells transfected with the pre-miR negative control (Figure 4B,C, respectively).

Based on such data, the upregulation of miR-146a, and the reduced expression of its targets, validated successful transfection. Moreover, because TRAF6 and IRAK1 are negative regulators of toll-like receptor signaling and NF-κB [72,73], we anticipated a beneficial influence on the ALS iAstrocyte reactive profile. In this context, we next investigated the effect of miR-146a upregulation on a set of common markers associated with the astrocyte aberrant phenotype in ALS [20,21], either in the ALS2 or the ALS7 iAstrocytes.

**Figure 5:**
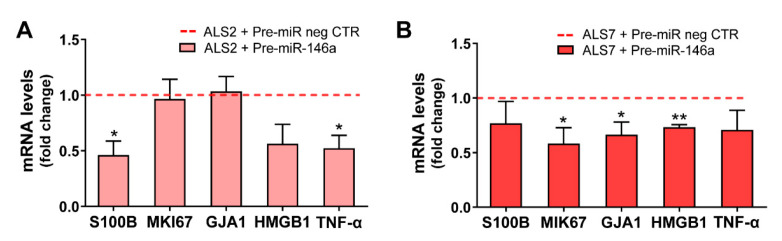
Overexpression of miRNA(miR)-146a in ALS-iAstrocytes exhibiting miR-146a downregulated levels differently reduces aberrant- and inflammatory-related markers in ALS2 and ALS7 cells. ALS patient fibroblasts were directly transdifferentiated into induced neural progenitor cells, and subsequently differentiated into induced astrocytes (iAstrocytes) for 7 days in vitro. Transfection with the pre-miR-146a or with the pre-miR negative control (pre-miR neg CTR) was performed in iAstrocytes from the mSOD1-ALS2 and sALS-7 cases. Expression of S100 calcium-binding protein B (S100B), MKI67 (gene that encodes for the proliferation marker Ki-67), gap junction alpha-1 (GJA1, gene that encodes the protein connexin 43 in humans), high mobility group box protein 1 (HMGB1) and tumor necrosis factor alpha (TNF-α) were assessed in iAstrocytes from (**A**) the mSOD1-ALS2 and (**B**) sALS7 patients, using RT-qPCR. Results are mean values ± SEM fold change vs. respective cells transfected with pre-miR neg CTR (red dashed line) from at least four independent experiments. * *p* < 0.05 and ** *p* < 0.01, paired Student’s *t*-test. ALS—amyotrophic lateral sclerosis; sALS—sporadic ALS; mSOD1—mutant superoxide dismutase 1.

Interestingly, the alterations produced in each of these cell lines after the transfection with the pre-miR-146a, relative to the pre-miR negative control, were revealed to be complementary (Figure 5). Consequently, we found a decrease in the gene expression of S100B and TNF-α using pre-miR-146a treatment on mSOD1-ALS2-iAstrocytes (*p* < 0.05, Figure 5A). However, in the case of ALS7, the upregulation of miR-146a with its mimic reduced the gene expression level of MIK67 (encoding for Ki-67), GJA1 (encoding for Cx43) and HMGB1 in ALS7 (at least *p* < 0.05, Figure 5B); these results support the heterogeneous presentation and progression patterns of ALS disease. 

**Figure 6:**
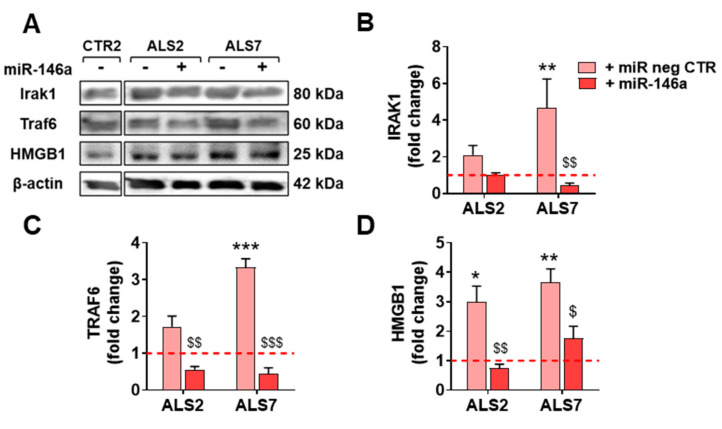
Transfection with pre-miR-146a reduces the expression of TRAF6 and IRAK1 proteins, which are known targets of microRNA(miR)-146a, as well as of the alarmin HMGB1. ALS patient fibroblasts were directly transdifferentiated into induced neural progenitor cells, and subsequently differentiated into induced astrocytes (iAstrocytes, iAst) for 7 days in vitro. For miR-146a upregulation, mSOD1-ALS2 and sALS7 iAst were transfected with pre-miR-146a. Cells transfected with the pre-miR negative control (pre-miR neg CTR) were used as controls. (**A**) Representative results of Western blot analysis and quantification of (**B**) TRAF6, (**C**) IRAK1, and (**D**) HMGB1 protein expression levels. Representative results from one blot in which all samples were applied in the same gel with equal protein concentration, and performed under the same conditions, are shown. Results are mean values ± SEM fold change vs. CTR2 cells transfected with pre-miR neg CTR (red dashed line), from three independent experiments. * *p* < 0.05; ** *p* < 0.01; *** *p* < 0.001 vs. non-ALS, one-way ANOVA followed by Dunnet’s post hoc test, or ^$^ *p* < 0.05; ^$$^ *p* < 0.01, ^$$$^ *p* < 0.001 vs. respective cells transfected with pre-miR neg CTR, one-way ANOVA followed by Bonferroni’s post hoc test. ALS—amyotrophic lateral sclerosis; HMGB1—high mobility group box protein 1; IRAK1—interleukin 1-receptor-associated kinase 1; mSOD1—mutant superoxide dismutase 1; sALS—sporadic ALS; TRAF6—TNF-receptor-associated factor 6.

We next investigated whether the decreased levels previously encountered for the TRAF6 and IRAK1 gene targets of miR-146a were reproduced at their protein levels. As depicted in Figure 6, the treatment with pre-miR-146a significantly decreased these proteins towards control levels (at least *p* < 0.05, except for IRAK1 in the ALS2 cells), reinforcing the success of the pre-miR-146a transfection and its benefits. We additionally assessed whether the regulation of miR-146a was translated into the normalization of the alarmin and pro-inflammatory marker HMGB1, whose gene expression was downregulated by pre-miR-146a—as shown above—mainly in the case of ALS7. As depicted in Figure 6, the treatment of ALS2 and ALS7 iAstrocytes with pre-miR-146a also reverted upregulated HMGB1 protein levels toward normal ones, attesting its modulatory efficacy. 

Together, our data support that the set of reactive astrocyte markers largely identified in ALS models [20,36,40,43] is successfully normalized by the pre-miR-146a regulation, contributing to the recovery of the neuroprotective phenotype of ALS iAstrocytes—despite the effect being exerted by different pathological signaling pathways—thus supporting the distinct reactive signatures in mSOD1-ALS2 and sALS7 samples.

### 3.5. Transfection of ALS2 and ALS7 iAstrocytes with Pre-miR-146a Successfully Enhances Its Expression Levels in the Secretome, without Modifying miR-155 Paracrine Signaling

As indicated earlier, soluble factors and molecules packaged in sEVs contribute to secretome-mediated cell pathogenicity [74,75], but also to regeneration [76,77,78]. We have previously shown that the secretome from mSOD1 mouse cortical astrocytes modulated with pre-miR-146a had neuroprotective properties [43]. Neurobiological differences between mice and humans seem to be the main cause of failed treatments in neurodegenerative diseases based on preclinical experiments in animal models [79,80]. Therefore, human stem cells, secretomes and sEVs became promising tools in clinical translation and regenerative medicine [77,81,82,83]. Here, we aimed to investigate whether the neuroprotective benefits of miR-146a upregulation in defective mSOD1 cortical mouse astrocytes are also observed after regulating its levels in depleted iAstrocytes with translation to their secretome, as a potential therapy for precision medicine. 

**Figure 7:**
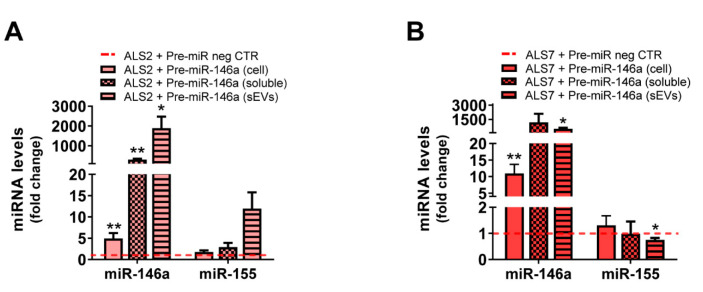
Transfection of pre-miR-146a in depleted ALS2 and ALS7 iAstrocytes leads to its upregulation in their secretome, with minimal changes in miR-155. ALS patient fibroblasts were directly transdifferentiated into induced neural progenitor cells, and subsequently differentiated into induced astrocytes (iAstrocytes) for 7 days in vitro. Transfection with the pre-miR-146a or with the pre-miR negative control (pre-miR neg CTR) was performed in iAstrocytes from the mSOD1-ALS2 and sALS-7 cases. Expression of microRNA(miR)-155 and miR-146a was assessed in iAstrocytes differentiated from the (**A**) mSOD1-ALS2 and (**B**) sALS7 patients, as well as in their secretome, either sEV-free (soluble) or in sEVs isolated from the extracellular media using RT-qPCR. Results are mean values ± SEM fold change vs. matched pre-miR neg CTR (red dashed line) from at least three independent experiments. * *p* < 0.05 and ** *p* < 0.01, paired Student’s *t*-test. ALS—amyotrophic lateral sclerosis; sALS—sporadic ALS; mSOD1—mutant superoxide dismutase 1; sEVs—small extracellular vesicles.

For that, we assessed whether the transfection of mSOD1-ALS2 and sALS7 with pre-miR-146a was recapitulated at the secretome levels of miR-146a—either in the sEV-free or in sEV secretome fractions—expecting to counteract the reduced values we previously found in iAstrocyte secretomes (Figure 3A,B). This modulation in the depleted ALS2 and ALS7 iAstrocytes led to its increased levels in cells, sEV-free secretomes, and sEVs in the first case (Figure 7A, at least *p* < 0.05), and only in cells and sEVs in the latter one (Figure 7B, at least *p* < 0.05) due to a slightly higher variation in the expression of miR-146a in the ALS7 sEV-free secretome, than in ALS2. 

miR-146a and miR-155 were shown to mediate neuroinflammatory responses [84] and miR-155 to be increased in the microglia from ALS models, without interfering in the expression levels of miR-146a in the mSOD1 mice, as demonstrated by Butovsky et al. [42]. However, to be sure that our modulation with pre-miR-146a did not contribute to the upregulation of miR-155, which was found elevated in the spinal cord of fALS and sALS patients [85] and associated with decreased mSOD1 mouse survival [42], we decided to investigate whether changes were produced on miRNA-155 by the pre-miR-146a. No differences (ALS2), or a small decrease only in sEVs (*p* < 0.05, ALS7), were noticed for miR-155 expression levels, which remained close to the values of non-treated cells (Figure 7A,B).

### 3.6. Upregulation of Depleted miR-146a in ALS2 and ALS7 iAstrocytes Diversely Contributes to the Recovery of Autophagic, Synaptic, and Axonal Transport Dynamics in NSC-34 MNs

Previous studies showed that miR-146a transfection in stem cells produces a secretome with reparative therapeutic paracrine molecules [86]. We also observed beneficial results when cortical astrocytes, defective in miR-146a, were treated with its mimic, supporting the recovery of the neuroprotective phenotype and the restoration of homeostatic paracrine signaling [43]. Indeed, ALS astrocytes were shown to contribute to MN degeneration [18,19,20,86] and the regulation of miR-146a expression in astrocytes, to have a protective role mediated by their secretome [43]. In conformity, based on data from previous experiments, and having the opportunity to work with astrocytes differentiated from ALS patient somatic cells, we anticipated that the pre-miR-146a treatment in the mSOD1-ALS2 and sALS7 iAstrocytes would modulate their secretome-mediated toxicity to MNs. For that, in this section we performed co-cultures of such iAstrocytes with the NSC-34 MN-like cells, for 72 h, based on our extensive experience with these cells [20,43,45,54].

**Figure 8:**
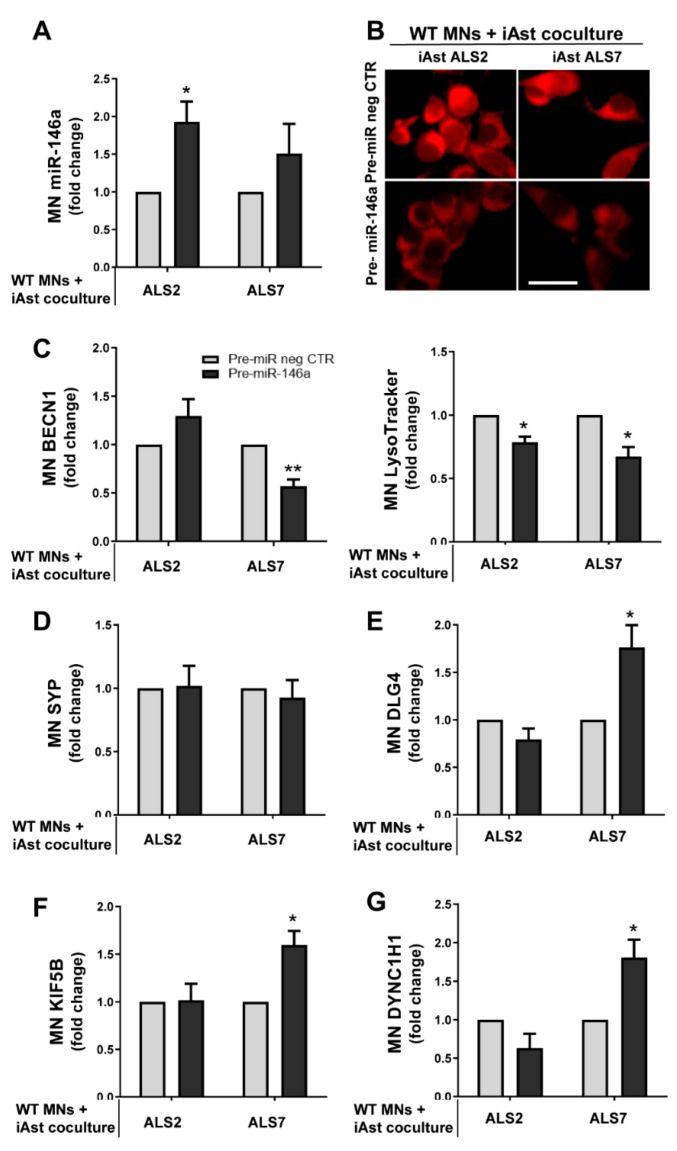
Pre-miR-146 treatment in ALS2 and ALS7 iAstrocytes leads to its upregulation in NSC-34 motor-neuron (MN)-like cells after co-culturing, while differently regulating the autophagic processes, synaptic protein dynamics and axonal transport in the target cells. ALS patient fibroblasts were directly transdifferentiated into induced neural progenitor cells, and subsequently differentiated into induced astrocytes (iAstrocytes) for 7 days in vitro. mSOD1-ALS2 and sALS7 iAstrocytes transfected with pre-miR-146a were co-cultured with WT NSC-34 MNs for 72 h. (**A**) Assessment of miRNA(miR)-146a expression in NSC-34 MNs after co-culture with ALS2 and ALS7 using RT-qPCR; (**B**) representative fluorescence images of WT MNs loaded with LysoTracker Red after being co-cultured with pre-miR-146a-treated iAstrocytes; Evaluation of (**C**) BECN1 (gene that encodes for beclin-1), (**D**) SYP (gene that encodes for the pre-synaptic protein synaptophysin), (**E**) DLG4 (gene that encodes for the post-synaptic protein 95, PSD95), (**F**) KIF5B (gene that encodes for kinesin-1), and (**G**) DYNC1H1 (gene that encodes for dynein) in MNs cultured with the miR-146a modulated ALS2 and ALS7 iAstrocytes using RT-qPCR. Results are mean values ± SEM fold change vs. WT MN co-cultured with matched pre-miR negative control (pre-miR neg CTR) from at least four independent experiments. * *p* < 0.05 and ** *p* < 0.01, paired Student’s *t*-test. ALS—amyotrophic lateral sclerosis; mSOD1—mutant superoxide dismutase 1. Scale bar represents 40 µm.

First, we assessed whether the pre-miR-146a treatment in ALS iAstrocytes was reflected in its expression in MNs after the co-culture. We noticed that miR-146a upregulation occurred in MNs treated with both the mSOD1-ALS2 and sALS7 iAstrocytes, but only significantly in the first case (*p* < 0.05, Figure 8A).

Such findings may derive from the higher content of miR-146a that we observed in sEVs from the modulated iAstrocytes ALS2 than in those from the ALS7 cells (Figure 7). Whether it differently counteracted MN degeneration was evaluated in our next step. For that, we assessed parameters related with the autophagic, synaptic and axonal transport processes that were shown to be dysregulated in neurodegeneration, including in ALS models and in our previous publications [20,40]. Autophagy was demonstrated to be upregulated in ALS MNs and to contribute to neurodegeneration [87]. In a first look, when globally assessing the influence that astrocytes exert on MNs, we observed that the healthy astrocytes showed a propensity to decrease beclin-1 and to increase synaptic and axonal transport-related genes. No alterations were caused in beclin-1 by the pathological astrocytes; however, they induced synaptophysin and depressed PSD-95—thus increasing inflammation and axonal damage [88] and rendering synapses more susceptible to insults [89], respectively—though data were not statistically significant. These alterations were tentatively reversed by modulation with pre-miR-146a, mainly in the ALS7 case for the axonal transport and PDD-95, when the folds were performed relatively to the MNs alone (Supplementary Figure S5). 

We then expressed the ALS2 and ALS7 data in folds vs. the effect of pre-miR neg CTR in the co-culture of WT MNs with iAstrocytes from the CTR2 cell line. We found reduced levels of LysoTracker (marker of lysosomes and autophagosomes) in MNs co-cultured with both miR-146a-treated ALS iAstrocyte lines (Figure 8B, *p* < 0.05). The other one was the BECN1 gene, encoding for beclin-1, the protein considered to be essential for autophagy [90]. We observed that it was significantly downregulated in the MNs co-cultured with the modulated ALS2 iAstrocytes (*p* < 0.01, Figure 8C). These data suggest that the upregulation of miR-146a in iAstrocytes leads to a reduction in MN autophagy by paracrine signaling. 

Synaptic failure is another important issue in ALS, and a predominant post-synaptic impairment has been described [91]. In conformity, we did not observe alterations in the pre-synaptic synaptophysin (encoded by the SYP gene) (Figure 8D); however, the levels of the DLG4 gene that encodes for PSD95 were increased in WT MNs upon exposure to pre-miR-146a-treated ALS7 iAstrocytes (*p* < 0.05), while remaining unchanged in the presence of ALS2 cells (Figure 8E). Finally, another important aspect of ALS is the disruption of axonal transport, due to the downregulation of dynein (retrograde) and kinesin-1 (anterograde) in degenerating ALS MNs [92,93]. In this context, we observed that miR-146a upregulation in sALS7 iAstrocytes also led to increased KIF5B (gene that encodes for kinesin-1) and DYNC1H1 (gene that encodes for dynein) (*p* < 0.05 for both, Figure 8F,G), but the same was not found in the experiments with the ALS2 iAstrocytes. The enhanced benefits of ALS7 iAstrocytes may derive from the more depressed levels of miR-146a in these cells than in those of ALS2, thus increasingly benefiting from miR-146a upregulation and the higher efficiency of the transfection in ALS7 iAstrocytes, together with a more effective targeting of both IRAK1 and TRAF6, compared to ALS2.

Collectively, these data validate that the modulation of astrocyte neurotoxic effects may implicate diverse protective mechanisms underlined by different pathways; this is relevant in patient stratification towards personalized medicine for ALS, and in the beneficial effects of multitarget strategies.

## 4. Discussion

Advanced experimental models of ALS are key to clarifying the complexity of the mechanisms of the human disease and to test new target-driven therapeutic approaches. ALS rodent models that only reflect particular aspects of ALS pathogenicity are not suitable to explore the sporadic forms of the disease and lack the ability for translation into the clinic [94]. Moreover, since ALS is a multifactorial disease and reveals variable pathogenesis among patients, it is hard to model because glial cells besides neurons are involved, which may be the main reason for the recurrent failure of clinical trials [4,5]. Moreover, substantial heterogeneity in clinical presentation urgently requires a better stratification of patients in subpopulations to accelerate disease understanding, develop drug trials and translate to clinical care [95]. Therefore, iPSC-derived neural cells and direct-cell-type conversion from patient somatic cells (circumventing the rejuvenated identity of iPSC-derived neural cells), are promising in representing patient-specific signatures, and also have potential for disease modeling, drug development and cell-derived regenerative therapies [96,97]. Lately, directly converted astrocytes were shown to retain the ageing signatures of patient fibroblasts while better recapitulating the disease state and individual profiling [19,26,98]. Functional tests in transdifferentiated iAstrocytes were revealed to be comparable to native brain astrocytes. Astrocytes were shown to release toxic factors and to have a key role in MN degeneration in both fALS and sALS patients [6,7,99,100]. Astrocyte-aberrant reactive features involving a decrease in GFAP and GLT-1 levels, an increased proliferative capacity, and an elevated expression of the Cx43 and S100B signature [20,21], together with inflammatory astrocytic markers [36,61], were identified in rodent and human ALS models. However, whether these markers are differently presented by fALS and sALS iAstrocytes was never investigated. Therefore, in the present study, we intended to explore the iAstrocyte signatures generated from seven fALS and sALS patients (three males and four females) for known markers of phenotype aberrancies and deregulated miRNA expression, either intracellularly or in their secretome. Because our previous studies identified downregulated miR-146a in cortical astrocytes isolated from the mSOD1 mice [20,40], and homeostatic benefits on MNs and microglia by pre-miR-146a upregulation [43], we looked for this special fingerprint and the resultant effects using its modulation in patient-derived iAstrocytes.

We observed a marked loss of HB9/GFP-positive MNs after exposure to directly converted iAstrocytes, either from mSOD1-ALS or sALS patients. This effect is in line with previous studies from Meyer et al. [19] and Haidet-Phillips et al. [18] showing that both mSOD1-ALS and sALS-derived astrocytes, or those isolated from the spinal cord tissue, are toxic to MNs. Interestingly, the transplantation of iPSC-derived ALS astrocytes was previously shown to also cause neuronal degeneration [101]. The less neurotoxic iAstrocytes were those generated from the youngest patients, usually presenting the bulbar form of the disease [102].

Besides the neurotoxic properties, the reactive profile of ALS iAstrocytes has been scarcely investigated. We found a reduction in GFAP expression exclusively in iAstrocytes from the two mSOD1-ALS patients, though other studies have pointed to elevated levels [103]. Such a reduction was previously identified by us and others in astrocytes from mSOD1 rodents [20,21], or after transfection with mSOD1 [104], and was shown to accelerate disease progression [105]. Elevated levels of GFAP were also not found in the blood of mSOD1 mice [106]. It was recently suggested that this aspect should not be considered, per se, an absolute marker of reactivity, or to represent cell altered functions when increased [107].

Cx43 upregulation was observed in the iAstrocytes from one mSOD1-ALS (ALS2) and from three sALS patient cell lines. Cx43 is a marker of astrocyte reactivity and astrogliosis [108], which was found to be enhanced in both fALS and SALS patients, as well as in mSOD1 mouse astrocytes, which contributed to MN toxicity [20,21,60]. The Cx43 increase only in female samples is in line with studies showing its sex-dependent expression [109,110]. Still accounting for the aberrant phenotype, and the most predominant marker among those we assessed, was the marked proliferative capacity in iAstrocytes derived from all mSOD1-ALS (except ALS1) and sALS patients. 

Also linked to astrocyte phenotypic reactivity in ALS is the dysregulation of miRNAs associated with inflammatory processes [40,111]. miRNAs are small, non-coding RNAs that travel in sEVs or form complexes with proteins when solubilized in the secretome [37]. The decreased miR-181b expression observed in the iAstrocytes of one mSOD1-ALS and one sALS patient may be associated with their reactive and pro-inflammatory profile, since this miRNA acts as a negative regulator of astrogliosis, proliferation and inflammatory response [112,113]. Regarding miR-155 and miR-146a, their alterations diverged among patients, while no significant differences were observed in miR-21. Upregulated miR-155 was noticed in the iAstrocytes of one mSOD1-ALS and two sALS cases and, as far as we know, only described in ALS spinal microglia and astrocytes, as well as in the skeletal muscle of patients [40,42,114], despite being observed in mouse cortical astrocytes exposed to amyloid beta fibrils [115]. miR-146a was elevated in one mSOD1-ALS and one sALS case and downregulated in one mSOD1-ALS and two sALS cell lines. In other studies, depressed miR-146a levels were observed in the cerebral cortex of mSOD1 mice in pre-symptomatic and symptomatic stages, as well as in cortical astrocytes, but found upregulated in the spinal tissue of the same animals [20,36]. miR-146a overexpression in this region was shown to be associated with MN loss in spinal atrophy [116]. Variable patterns were found in spinal cord and muscle biopsies [42,85,114], and reduced expression values were identified in the CSF and serum of sALS patients [117,118]. This variability may derive from CNS regional differences in ALS astrocyte phenotypes and may eventually be promising in discriminating the bulbar and spinal forms of the disease, when using iAstrocytes as a tool to stratify patients. Dependence on such potential targets and associated specific modulation of ALS astrocytes should be considered in the translation from the patient to the lab, and back to the clinic. 

sEVs are recognized to carry miR-146a and miR-155 [43,119], and to be players in ALS disease dissemination [120]. As such, we selected iAstrocytes from two mSOD1 patients (ALS2 and ALS3) and another two sALS cases (ALS6 and ALS7) that showed intracellular dysregulation of miRNA-146a and upregulation of miR-155 (only the ALS7 case). Our aim was to explore the representation of miR-146a and miR-155 in the cell secretomes, either as EV-free soluble material or as part of sEV cargo. A reduction in miR-155 below control values was observed in all the assessed cell lines, either in sEVs or as soluble miRNAs, thus compromising paracrine signaling. In conformity, a lower ability to immunoregulate their targets in the recipient cells and a compromised inflammatory response in a pathological circumstance are expected to occur due to the reduced levels of miR-155 in the secretome. Such dysregulation, previously found in our laboratory in sEVs from ALS cortical and spinal astrocytes, may compromise the brain immune system [121]. Clarification of these findings requires further studies in ALS human models using tricultures of neurons, astrocytes and microglia, spheroids, or organoids. In fact, the targeting of increased miR-155 in spinal microglia was found to attenuate disease progression in mSOD1 adult mice, and an antibody is being developed by miRagen Therapeutics, Inc; however, the effects on astrocytes and disease state were never investigated, and even less investigation was conducted in the cerebral cortex. It should be noted that this inhibition in astrocytes may result in harmful effects, as it may further contribute to decreasing the already low levels of miR-155 in the secretome. Our data reinforce that the stratification of patient sub-populations should precede the testing of such therapeutic strategies, since it may have either benefits or adverse consequences. In contrast with miR-155, circulating miR-146a was revealed to better recapitulate its levels in the cells. We found a higher secretion of miR-146a in sEV-free secretome or as part of sEV cargo in the case of the cell line overexpressing miR-146, but a decreased release into the secretome if originated from the iAstrocytes with depleted miR-146a levels. Such diverse results were not determined by differences in the number or size of the sorted sEVs and can be associated with cell decisions on mediating either active or passive release, according to the cellular metabolic requirements [39]. 

Because we were pioneers in highlighting the regulation of miR-146a for astrocyte neuroprotective effects in ALS models [43], we treated the most depleted mSOD1-ALS2 and sALS7 iAstrocytes with pre-miR-146a. Though we similarly succeeded in increasing miR-146a intracellular levels and downregulating its IRAK1 target, only the modulated sALS7 iAstrocytes showed a simultaneous reduction in the TRAF6, indicating differences in the cell pathological pathways, as observed in other published studies [122,123]. This finding is validated by the different pre-miR-146a benefits in the reactive/inflammatory signatures of ALS2 and ALS7, none of them shared by both. In ALS2, the modulation led to decreased S100B and TNF-α expression values, while in ALS7, it downregulated Ki67, Cx43 and HMGB1 expression levels. All of these are well-recognized markers of astrocyte phenotypic aberrancies and are shown to be upregulated in several ALS models [20,21,36,43,74,124,125]. Transfection with the miR-146a mimic in iAstrocytes from ALS2 and ALS7 patients presenting miR-146a depressed levels led to a marked elevation in soluble miR-146a in the secretome of ALS2 iAstrocytes, and an enrichment in sEVs from both treated cells; however, it only led to some minor changes in miR-155. These features corroborate previous findings using the miR-146a transfection in human adipose-derived stem cell to enrich its content in sEVs with benefits in modulating inflammation and enhancing tissue regeneration [86]. Similar data were observed in cortical ALS astrocytes, where transfection with pre-miR-146a led to a homeostatic secretome able to prevent MN degeneration and microglia activation [43]. 

With the switch in the reactive/inflammatory signature of ALS2 and ALS7 iAstrocytes by the regulation of miR-146a expression values toward control ones, and the enrichment of this miRNA in their secretome—mainly in sEVs—it was not unexpected to also observe an upregulation of miR-146a in the recipient NSC-34 MN-like cells after co-culture. This finding supports the propagation of miRNAs from donor to target cells [43,45,126]. In conformity, some neuroprotective effects could be observed, as in other studies [127], though they were differently induced by either ALS or ALS7 iAstrocytes treated with pre-miR-146a.

Secretomes from both ALS2 and ALS7 iAstrocytes transfected with pre-miR-146a led to a reduction in LysoTracker Red staining in MNs, while sALS7 cells were unique in evidencing beclin-1 downregulation, which was found to be upregulated in postmortem tissues derived from sALS and fALS cases [128]. Therefore, this could represent a neuroprotective effect considering that enhanced autophagy in ALS MNs was shown to promote neurodegeneration and that beclin-1 reduction was associated with the increased lifespan of the mSOD1 mice [129]. Although no changes were noticed in the pre-synaptic synaptophysin in any of the ALS iAstrocytes after co-culture with MNs, increased DLG4 gene expression that encodes for the postsynaptic PSD95 was found to be upregulated in the ALS7 experiments. Such data indicate the privileged MN functional benefits in this case, by protecting the synaptic failure attributed to the ALS disease [130]. In the same way, the increased expression of genes associated with the axonal transport proteins kinesin-1 (KIF5B) and dynein (DYNC1H1) in MNs, after co-culture with patient iAstrocytes, reinforces the benefits of miR-146a regulation in the depleted sALS7 cells. This represents an important consequence if we consider that the disruption of axonal transport in ALS is elicited by the phenotypic dysregulated astrocytes [131,132]. Again, we must emphasize that such response was not reproduced in the co-cultures of MNs with the miR-146a-treated mSOD1-ALS2 iAstrocytes, supporting patient subsets that do not respond to the same treatment [95]. 

Since only a small number of patients were studied, further works should consider a larger sample, though the low yield of the direct conversion technology and its complex steps still bring important limitations [19,29,61,62,63]. Despite this, our present study, highlights the use of directly converted iAstrocytes from ALS patients as an important tool for precision medicine. By stratifying patients, research using iAstrocytes may identify treatments that have adverse effects or that do not have efficacy in a particular patient, while helping in the selection of those who would most benefit from the developed target-specific and personalized medicine for ALS.

## 5. Conclusions

In summary, the present pilot study provides new evidence to support the variability between ALS patients and its recapitulation in their fibroblast-derived iAstrocytes. Importantly, differential dysregulation of GFAP, Cx43, Ki-67, miR-155, and miR-146a in sALS and mSOD1-iAstrocytes highlights their potential in identifying different signatures and stratifying patients. Further studies investigating the presence of such promising biomarkers in circulating exosomes expressing the astrocyte-linked glutamate aspartate transporter (GLAST) will open new perspectives for predicting ALS and monitoring ALS patients and their sub-populations in a non-invasive manner. We also confirmed that at least some of the patients manifest downregulated miR-146a in their somatic-derived astrocytes, and that its regulation towards steady-state values account for the recovery of the neuroprotective phenotype of these glial cells and restores functional homeostasis. This finding suggests that by targeting depleted miR-146a in pathological astrocytes with its mimic, we may be intervening in reducing the release of toxic factors into the secretome, in addition to mediating miR-146a extracellular signaling cascades. Actually, our previous data showed that miR-146a replenishment in depleted astrocytes protects MNs from degeneration and microglia from excessive activation [43]. Together with other biomarkers, miR-146a may have theragnostic value, allowing patient subtype stratification and treatment; meanwhile, its packaging in sEVs can lead to successful outcomes in ALS biomedical medicine. Patient and disease heterogeneity, limited knowledge on ALS pathophysiology, a lack of effective treatment, and an absence of robust biomarkers highlight the relevance of the present study with iAstrocytes derived from fALS and sALS patients and provide future directions for research validation.

## Figures and Tables

**Table 1:** Summary of the demographic information of the iAstrocyte-derived fibroblast lines from patients with amyotrophic lateral sclerosis and the controls used in this study.

Cell Line	Diagnosis	Mutation	Age at Biopsy (yrs)	Onset Type	Symptom Onset to Biopsy	Sex
**CTR1**	Non-ALS	-	42	-	-	Male
**CTR2**	Non-ALS	-	64	-	-	Female
**ALS1**	ALS	SOD1 ^1^	40	Lower limb	3 months	Male
**ALS2**	ALS	SOD1 ^2^	63	Lower limb	8 months	Female
**ALS3**	fALS	SOD1 ^3^	40	Unknown	Unknown	Male
**ALS4**	sALS	Unknown	29	Bulbar	2.7 Yrs	Male
**ALS5**	sALS	Unknown	62	Distal upper extremity	2.5 Yrs	Female
**ALS6**	sALS	Unknown	47	Distal upper extremity	1.75 Yrs	Female
**ALS7**	sALS	Unknown	69	Distal upper extremity	2.25 Yrs	Female

CTR—control; ALS—amyotrophic lateral sclerosis; sALS—sporadic ALS; fALS—familial ALS; SOD1—superoxide dismutase 1; Yrs—years. ^1^ D76Y mutation; ^2^ c.374A>G, p.Asp125 Gly; ^3^ A4V mutation.

## Data Availability

The data presented in this study are available on request from the corresponding authors.

## Supplementary Materials

The following supporting information can be downloaded at: https://www.mdpi.com/article/10.3390/cells11071186/s1, Table S1. List of antibodies used in the study, and respective suppliers; Table S2. List of primer sequences used in RT-qPCR; Figure S1. Settlement of retrovirus concentration for the transdifferentiation of fibroblasts in induced neural progenitor cells (iNPCs); Figure S2. Representative images of fibroblast transdifferentiation into iNPCs, differentiation into iAstrocytes and characterization, as well as of motor neuron differentiation from meSCs; Figure S3. Representative images of the co-cultures of GFP-positive HB9+ MNs with iAstrocytes. Figure S4. Transfection of ALS2 and ALS7 iAstrocytes with pre-miR-146a did not affect cell viability. Figure S5. Expression of motor-neuron associated markers after co-culture with astrocytes, either untreated or after miR-146a modulation, from non-ALS and ALS patients.
