# Supplementary material for: Neurotoxic Astrocytes Directly Converted from Sporadic and Familial ALS Patient Fibroblasts Reveal Signature Diversities and miR-146a Theragnostic Potential in Specific Subtypes
Source: Cells. 2022 Apr 1;11(7):1186. doi: 10.3390/cells11071186 (PMC8997588; doi:10.3390/cells11071186)
Supplement: Supplementary file 1 [file cells-11-01186-s001.zip › cells-1604800-supplementary.pdf]

cells

MDPI

Supplementary Materials

Neurotoxic Astrocytes Directly Converted from Sporadic and Familial ALS Patient Fibroblasts Reveal Signature Diversities and miR-146a Theragnostic Potential in Specific Subtypes

Cátia Gomes [1,2], Catarina Sequeira [1], Shibi Likhite [2], Cassandra N. Dennys [2], Stephen J. Kolb [3], Pamela J. Shaw [4], Ana R. Vaz [1,5], Brian K. Kaspar [2], Kathrin Meyer [2] and Dora Brites [1,5,*]

1 Research Institute for Medicines (iMed.ULisboa), Faculty of Pharmacy, Universidade de Lisboa, 1649-003 Lisbon, Portugal; catia.svgomes@gmail.com (C.G.); catseq95@gmail.com (C.S.); armvaz@ff.ulisboa.pt (A.R.V.)
2 The Research Institute, Nationwide Children's Hospital, Ohio State University, Columbus, OH 43205, USA; shibi.likhite@nationwidechildrens.org (S.L.); cassandra.dennys-rivers@nationwidechildrens.org (C.N.D.); brian.kaspar@nationwidechildrens.org (B.K.K.); kathrin.meyer@nationwidechildrens.org (K.M.)
3 Department of Neurology, The Ohio State University Wexner Medical Center, Columbus, OH 43205, USA; stephen.kolb@osumc.edu
4 Sheffield Institute for Translational Neuroscience (SITraN), University of Sheffield, Sheffield S10 2HQ, UK; pamela.shaw@sheffield.ac.uk
5 Pharmaceutical Sciences and Medicines, Faculty of Pharmacy, Universidade de Lisboa, 1649-003 Lisbon, Portugal
* Correspondence: dbrites@ff.ulisboa.pt; Tel.: +351 217946450

Table S1. List of antibodies used in the study and respective suppliers.

Primary antibody against:	Source	Species	Dilution used in: ICC	WB
Ki-67	Leica microsystems (Novocasta), KI67-CE	Rabbit	1:1500	---
CD44	Abcam	Mouse	1:1000	---
GFAP	AbCam, ab7260	Rabbit	---	1:500
Cx43		Rabbit	---	1:500
Oct3/4	Santa Cruz Biotechnology	Goat	1:200	---
Klf4	Santa Cruz Biotechnology	Rabbit	1:200	---
Sox2	Millipore	Rabbit	1:200	---
c-Myc	Santa Cruz Biotechnology	Rabbit	1:200	---
Irak1	Santa Cruz Biotechnology, sc-5288	Mouse	---	1:200
Traf6	Santa Cruz Biotechnology, sc-8409	Rabbit	---	1:200
HMGB1	Biolegend, 651402	Mouse	---	1:500
β-actin	Sigma, A5441	Mouse	---	1:5000
**Secondary antibody**	**Source**		**Dilution**	
Anti-mouse AlexaFluor 594	Invitrogen Corporation, A-11005		1:1000	---
Anti-rabbit AlexaFluor 488	Invitrogen Corporation, A-11008		1:1000	---
Anti-goat AlexaFluor 594	Invitrogen Corporation, A-21468		---	1:1000
IRDye® 800CW got anti-rabbit	Licor Cat No. 92632211		---	1:20000
IRDye® 680LT goat anti-mouse	Licor Cat No. 92668020		---	1:20000

Anti-mouse HRP	Santa Cruz Biotechnology, sc2005	---	1:5000
Anti-rabbit HRP	Santa Cruz Biotechnology, sc2004	---	1:5000

Cx43, connexin 43; GFAP, glial fibrillary acidic protein; HMGB1, high-mobility-group-box protein 1; ICC, immunocytochemistry; IRAK1, interleukin-1 receptor-associated kinase 1; Klf4, Krüppel-like factor 4; Oct3/4, POU transcription factor 3/4; TRAF6, TNF receptor associated factor 6; Sox2, SRY-related HGM-Box Gene 2; WB, western blot.

Table S2. List of primer sequences used in RT-qPCR.

Gene	Forward primer sequence	Reverse primer sequence
hsa TRAF6	5’-CCTTTGGCAAATGTCATGTGTG-3’	5’-CTCTGCATCTTTTCATGGCAA-3’
hsa IRAK1	5’-CTGGAAGGCAGAAAAGTTGG-3’	5’-TGTGACTCACGGCTGAACAC-3’
hsa S100B	5’-TGTAGACCCTAACCCGGAGG-3’	5’-TGCATGGATGAGGAACGCAT-3’
hsa MKI67	5’-TCCTTTGGTGGGCACCTAAGACCTG-3’	5’-TGATGGTTGAGGCTGTTCCTTGATG-3’
hsa Cx43 (GJA1)	5'-GTTCAATCACTTGGCGTGAC-3'	5'-AGTTGAGTAGGCTTGAAC-3'
hsa HMGB1	5'-CATCTCAGGGCCAAACCGATA-3'	5'-AGCAGACATGGTCTTCCACC-3'
hsa TNFα	5’-AACCTCCTCTCTGCCATC-3’	5’-ATGTTCGTCCTCCTCACA-3’
hss β-actin	5’-ATCCATGGTGAGCTGGCGGC-3’	5’-CAGAGCCTCGCCTTTGCCGA-3’
hss GAPDH	5’-CGCTCTCTGCTCCTCCTGTT-3’	5’-CCATGGTGTCTGAGCGATGT-3’
mus SYP	5’-GACGTTGGTAGTGCCTGTGA-3’	5’-GCACAGGAAAGTAGGGGGTC-3’
mus DLG4 (PSD95)	5’-GAGGCTGGCCAGTACAACAG-3’	5’-ACAGAAGAGCAGGCGGTCAG-3’
mus DYNC1H1	5’-GCCTCAGTCTCTGTCCCATC-3’	5’-AAGTCCTGGGGTAAGGTGCT-3’
mus KIF5B	5’-GGTCCTACAGTTGCCACCTA-3’	5’-AATGAAATACGCCAGGCCCA-3’
mus β-actin	5’-GCAGGAGTACGATGAGTCCG-3’	5’-ACGCAGCTCAGTAACAGTCC-3’

miRNA	Target sequence
**hsa-miR-181b-5p**	5’-AACAUUCAUUGCUGUCGGUGGGU-3’
**hsa-miR-21-5p**	5’-UAGCUUAUCAGACUGAUGUUGA-3’
**hsa-miR-155-5p**	5’-UUAAUGCUAAUCGUGAUAGGGGU-3’
**hsa-miR-146a-5p**	5’-UGAGAACUGAAUUCCAUGGGUU-3’
**U6**	Reference gene

TRAF6, TNF receptor associated factor 6; IRAK1, interleukin-1 receptor-associated kinase 1; S100B, S100 calcium binding protein B; MKI67, gene that encodes the proliferation marker Ki-67; GJA1, gap junction alpha-1 protein in human (also known as connexin 43 in mouse – Cx43); HMGB1, high-mobility-group-box protein 1; TNF-α, tumor necrosis factor alpha; SYP, synaptophysin; DLG4, gene that encodes the postsynaptic density protein 95 (PSD-95); DYNC1H1, gene that encodes for dynein; KLF5B, gene that encodes kinesin-1; miR, microRNA.

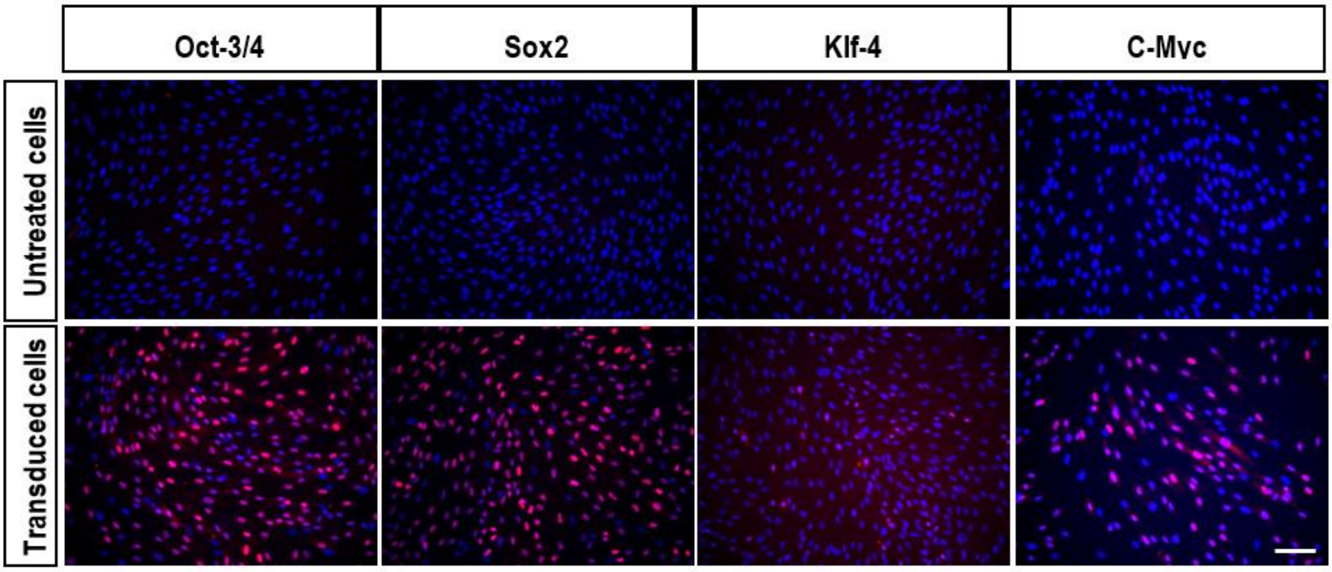

Figure S1. Settlement of retrovirus concentration for the transdifferentiation of fibroblasts in induced neuronal progenitor cells (iNPCs). Human fibroblasts were incubated overnight (~12h), individually, with different concentrations of each retrovirus carrying Kruppel-like factor 4 (Klf4), POU transcription factor Oct-3/4 (Oct3/4), SRY-related HMG-Box Gene 2 (Sox2) or c-Myc. After 2-3 days of recovery, cells were stained, by immunocytochemistry, with the antibodies against each factor. Representative images of the number of positive cells for each reprograming factor (red). Nuclei were stained with DAPI. Scale bar represents 50 μm.

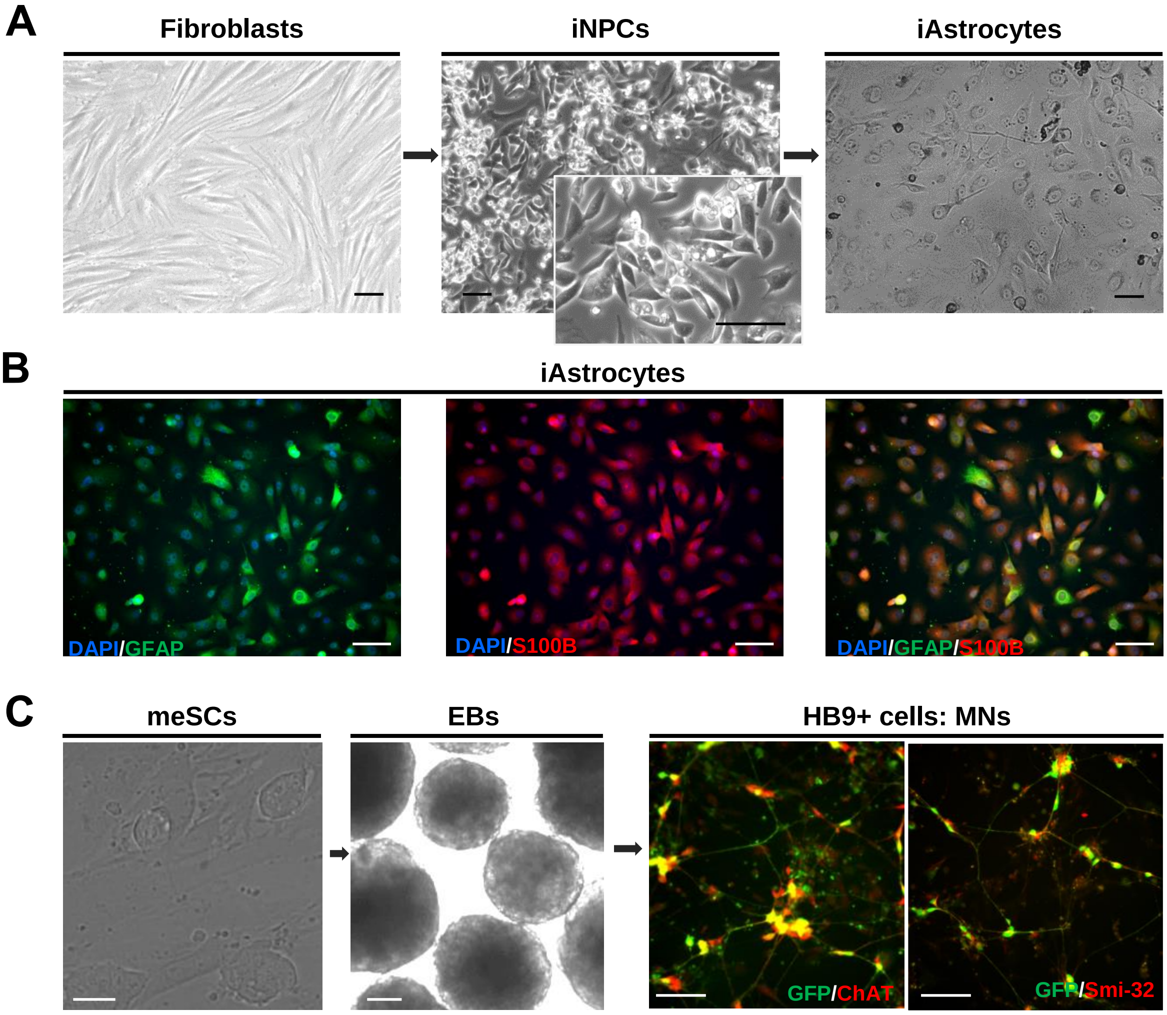

Figure S2. Representative images of fibroblast transdifferentiation into iNPCs, differentiation into iAstrocytes and characterization, as well as of motor neuron differentiation from meSCs. (**A**) Bright field representative images of the different culture stages in the direct conversion of human fibroblasts into induced neural progenitor cells (iNPCs) and their subsequent differentiation into iAstrocytes. Human fibroblasts were transduced with retroviral vectors carrying four reprogramming factors (Sox2, KLF4, Oct3/4, c-Myc). Within 6-10 days, cells exhibit marked morphological changes from a fibroblastic spindle like shape to a sphere-like form commonly seen in iNPCs. Subsequently, iNPCs were seeded in low concentration and maintained 6-7 days in astrocyte differentiation conditions. (**B**) Immunostaining characterization of iAstrocytes for the common markers glial fibrillary acidic protein (GFAP, in green), and S100B (in red). Cell nuclei were stained with DAPI (blue). (**C**) Representative images of the motor neuron (MN) differentiation protocol. Mouse embryonic stem cells (meSCs) expressing green fluorescent protein (GFP) under the MN-specific promoter HB9, cultured as embryoid bodies (EBs), were differentiated into MNs using retinoic acid (RA) and smoothened agonist (SAG). Differentiated MNs were immunostained with choline acetyltransferase (ChAT) and neurofilament protein Smi-32. The double staining of GFP with ChAT or Smi32 confirm the success of the differentiation method. Finally, after differentiation, EBs were sorted based on GFP levels using a FACSVantage/DiVa sorter. Scale bar represents 50 μm.

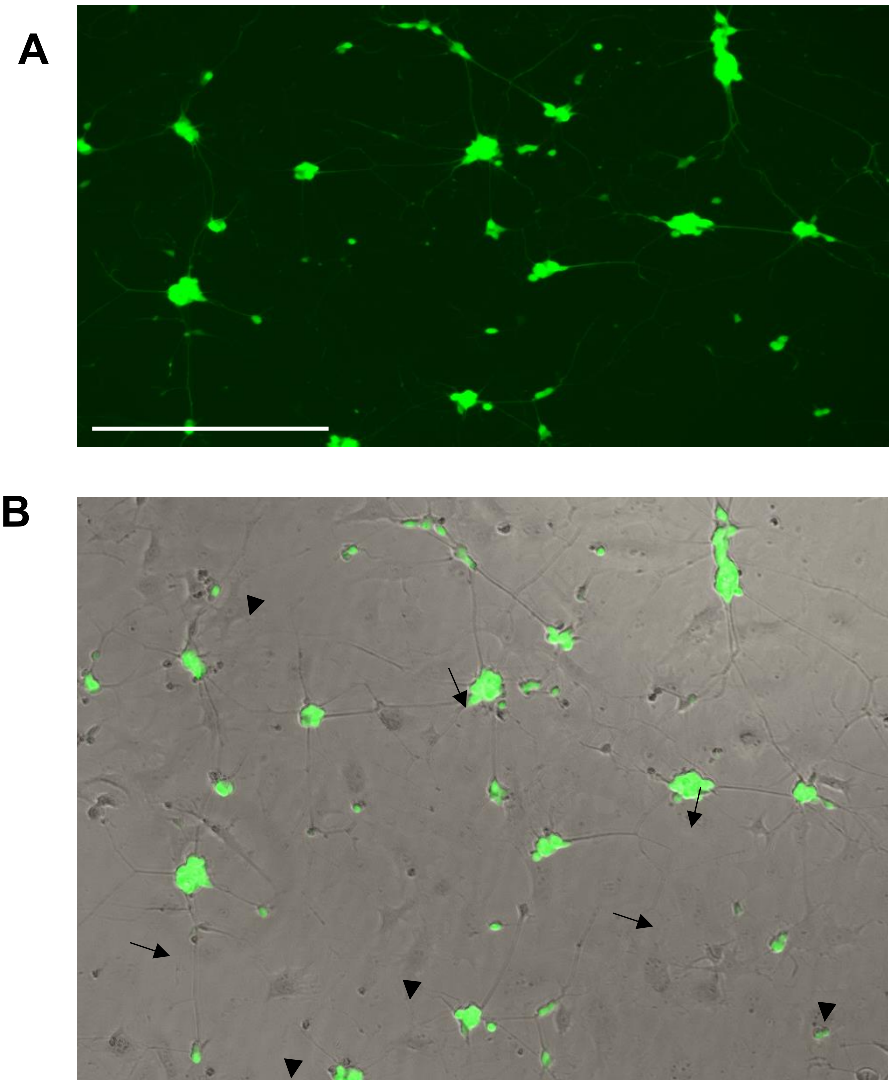

Figure S3. Representative images of GFP-positive HB9+ motor neurons (MNs) and iAstrocytes in co-culture. (**A**) Fluorescence image of MNs (arrow, green) and (**B**) merged fluorescence and bright-field image of astrocytes (arrowhead). Mixed neuron-astrocyte culture composed of around 50% of iAstrocytes and 50% GFP-positive MNs, each cell type at a density of 10,000 per well, as explained in Materials and Methods section. Scale bar represents 450 μm.

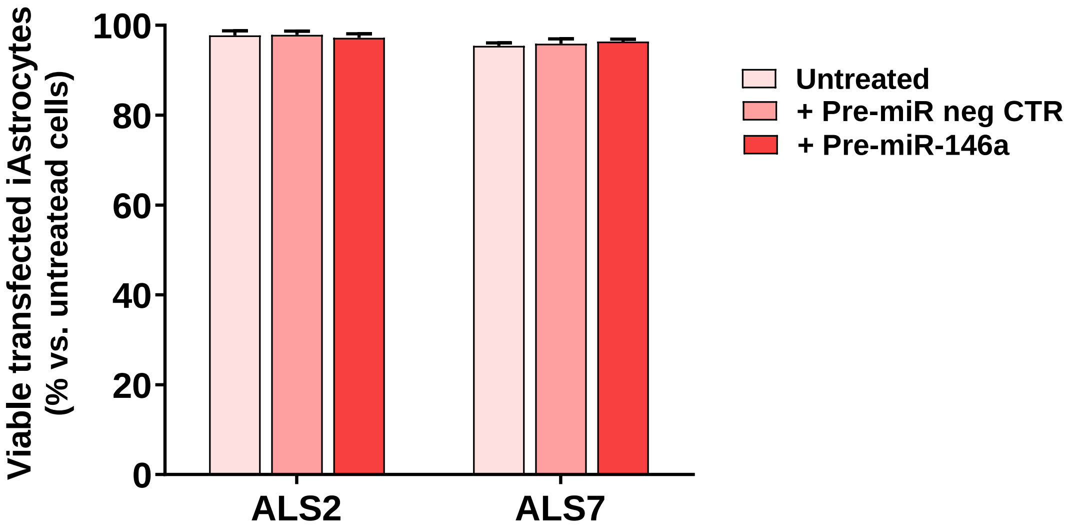

Figure S4. Transfection of ALS2 and ALS7 iAstrocytes with pre-miR-146a did not affect cell viability. ALS patient fibroblasts were directly transdifferentiated into induced neural progenitor cells (iNPCs), and subsequently differentiated into induced astrocytes (iAstrocytes) for 7 days *in vitro*. For miR-146a upregulation, the iAstrocytes from the SOD1-mutated ALS2 patient and from the sporadic ALS7 patient were transfected with pre-miR-146a. Viability of untreated and pre-miR-146a-treated iAstrocytes was assessed by Guava Nexin®, as previously described by us [53]. Results are mean percentage of viable cells ± SEM from two (ALS2) and three (ALS7) independent experiments.

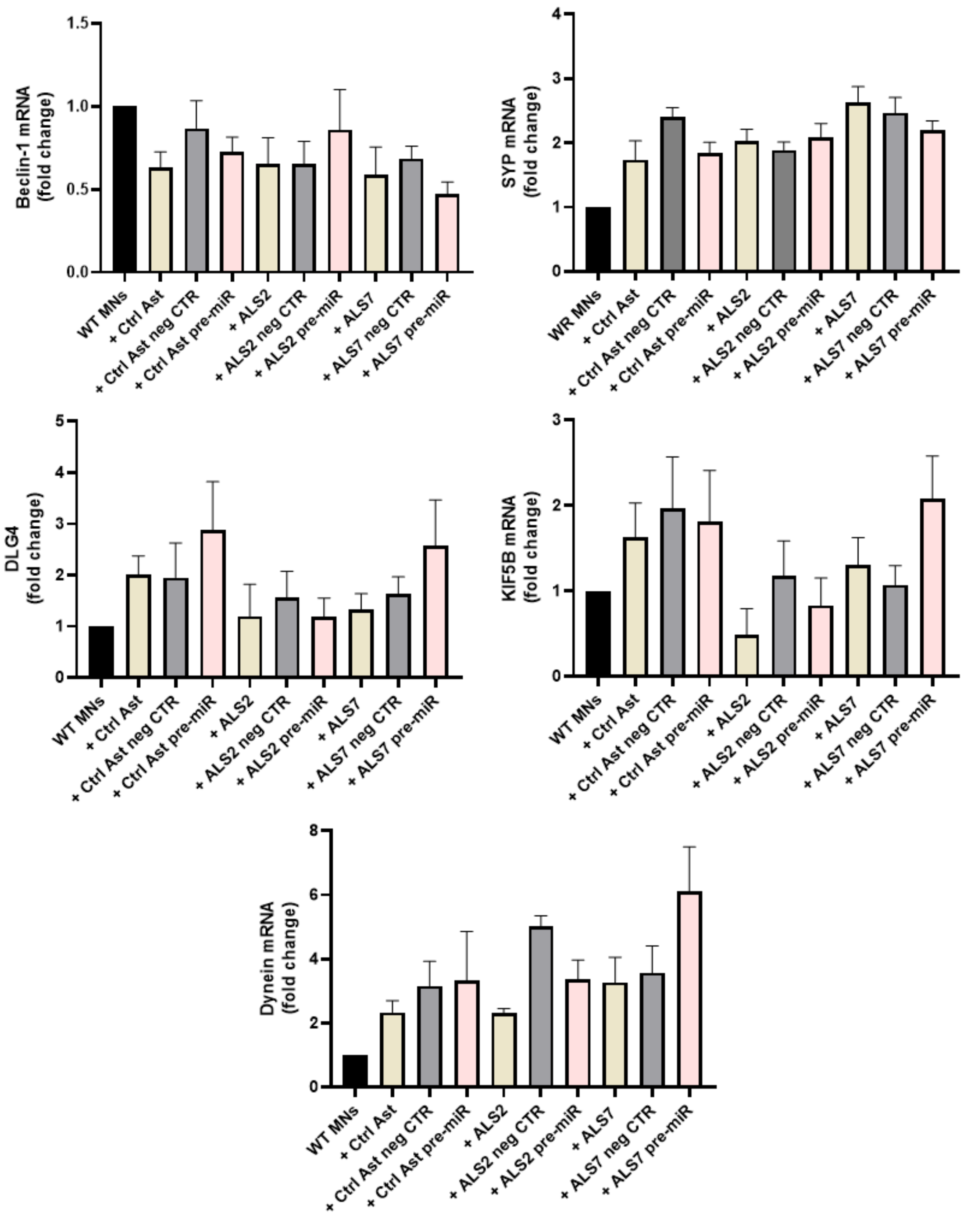

Figure S5. Expression of motor-neuron associated markers after co-culture with astrocytes, either untreated or after miR-146a modulation, from non-ALS and ALS patients. ALS patient fibroblasts were directly transdifferentiated into induced neural progenitor cells (iNPCs), and subsequently differentiated into induced astrocytes (Ast) for 7 days in vitro. Untreated (cream color) and pre-miR-146a-treated (pink color) astrocytes were co-cultured with WT NSC-34 MNs for 72 h (black). Evaluation of BECN1 (gene that encodes for beclin-1), SYP (gene that encodes for the pre-synaptic protein synaptophysin), DLG4 (gene that encodes for the post-synaptic protein 95, PSD95), KIF5B (gene that encodes for kinesin-1), and DYNC1H1 (gene that encodes for dynein) in MNs were assessed by RT-qPCR. Results are mean values ± SEM fold change vs. WT MNs alone. WT MNs, wild type motor neurons, from at least four independent experiments; Ctrl, Ast from non-ALS individuals; neg CTR, pre-miR negative control (grey color); ALS, amyotrophic lateral sclerosis; ALS2 and ALS7, tested ALS patients.
